# Graphene-Based One-Dimensional Terahertz Phononic Crystal: Band Structures and Surface Modes

**DOI:** 10.3390/nano10112205

**Published:** 2020-11-05

**Authors:** Ilyasse Quotane, El Houssaine El Boudouti, Bahram Djafari-Rouhani

**Affiliations:** 1 Laboratoire de Physique de la Matière et de Rayonnement (LPMR), Département de Physique, Faculté des Sciences, Université Mohammed I, 60000 Oujda, Morocco; q_ilyasse@yahoo.com; 2 Institut d’Electronique, de Microélectronique et de Nanotechnologie (IEMN), UMR CNRS 8520, Département de Physique, Université de Lille, 59655 Villeneuve d’Ascq, France; bahram.djafari-rouhani@univ-lille.fr

**Keywords:** graphene, phonon, crystal, surface mode, resonant mode

## Abstract

In this paper, we provide a theoretical and numerical study of the acoustic properties of infinite and semi-infinite superlattices made out of graphene-semiconductor bilayers. In addition to the band structure, we emphasize the existence and behavior of localized and resonant acoustic modes associated with the free surface of such structures. These modes are polarized in the sagittal plane, defined by the incident wavevector and the normal to the layers. The surface modes are obtained from the peaks of the density of states, either inside the bulk bands or inside the minigaps of the superlattice. In these structures, the two directions of vibrations (longitudinal and transverse) are coupled giving rise to two bulk bands associated with the two polarizations of the waves. The creation of the free surface of the superlattice induces true surface localized modes inside the terahertz acoustic forbidden gaps, but also pseudo-surface modes which appear as well-defined resonances inside the allowed bands of the superlattice. Despite the low thickness of the graphene layer, and though graphene is a gapless material, when it is inserted periodically in a semiconductor, it allows the opening of wide gaps for all values of the wave vector $k_{//}$ (parallel to the interfaces). Numerical illustrations of the band structures and surface modes are given for graphene-Si superlattices, and the surface layer can be either Si or graphene. These surface acoustic modes can be used to realize liquid or bio-sensors graphene-based phononic crystal operating in the THz frequency domain.

## 1. Introduction

Graphene is a two-dimensional (2D) hexagonally bonded flat sheet of monolayer carbon atoms, which are tightly packed in a honeycomb crystal lattice. It was first described theoretically by Wallace decades ago [1]. It can be rolled to form one-dimensional nanotubes [2], wrapped to form zero dimensional fullerenes [3] or stacked to form three-dimensional graphite. The study of graphite was restricted to fewer layers until 2004 when Geim and Novoselov [4,5] isolated for the first time an Single-layer of graphene from graphite for which they were awarded the Nobel Prize in Physics in 2010.

Among Graphene’s unique properties, there is the peculiar collective behavior of electrons. The interaction between electrons and the honeycomb lattice ensures that electrons are governed by the Dirac equation and behave as if they had absolutely no mass [6], and it is considered as a gapless semiconductor material as well as an ideal 2D electron gas system. Due to its superior transport properties along with its outstanding mechanical and thermal properties, intensive theoretical and experimental research has been conducted in recent years on the important and unique physical properties of electron-based graphene systems [7]. Besides its distinctive properties, graphene has shown excellent performance in all aspects, such as: nano-electronics [8,9], spintronic [10], optical, photonic and optoelectronic [11], mechanics with high Young’s modulus [12] and negative Poisson’s ratio [13], high mobility at ambient temperature [14], high factor of light transmission [15], and a thermal conductivity that can reach 5000 W m ^−1^K^−1^, several times greater than that of diamond [16,17]. Indeed, graphene has already been used to realize high frequency electronic devices and field effect transistors with high speeds [18], single-electron transistor [19], soft touch screens [20], and transparent electrodes for visible [21] and infrared [22] optoelectronic devices. Graphene is also known for its abundant potential applications in sensing devices [23], such as biosensor based on graphene where it has shown the ability of detection with high sensitivity [24]. In addition, another way to exploit the properties of graphene for useful applications would be to combine graphene sheets into a complex material structure [25]. It is known that, for heterostructure materials, the modulation of physical properties across different material layers can lead to new physical and electrochemical properties in such systems [25,26]. Such composite structures made of different multilayers have been widely applied in the realization of semiconductor heterostructures, like superlattices (SLs) and quantum wells for the design of small size electronic systems [27]. In addition, the generation and excitation of surface waves using graphene-based grating for one-dimensional photonic structures have been demonstrated experimentally [28]. Moreover, the outstanding graphene unique properties in all aspects have attracted tremendous interest like their capability of supporting different types of band structures and surface modes called optical Tamm states in graphene-dielectric multilayers [29,30,31]. In addition, graphene based metamaterials have been designed for applications as absorbers [32] and on-chip integrated photonic devices with potential applications [33].

Thus far, the study of the acoustic properties of multilayered materials has been the subject of intensive theoretical and experimental studies [34]. A thorough understanding of phonon behavior in these advanced material systems has been achieved [35,36]. One of the main acoustic properties of an SL is the possibility to realize a Bragg mirror of sound waves phonons or long wavelength. In fact, different acoustic devices based on multilayers, such as mirrors, filters, and resonators have been experimentally realized in the high-frequency domain [37,38,39].

In recent years, the coherent generation of acoustic and electric waves in the terahertz frequency range from diverse semiconductor-based systems achieved a huge progress [20]. In Ref. [40], they observed that the application of an external electrical bias to a weakly coupled semiconductor SL made of GaAs-AlAs gives rise to an increasing in the amplitude of the coherent hypersound oscillations generated by a femtosecond lasers, as well as the spectral narrowing of the phonon mode of the SL with a frequency of 441 GHz. These results show that the THz acoustic based on the sound amplification through stimulated emission of phonons leads to an essential step to generate a coherent THz sound (saser) and other hypersonic devices. The study of THz sound waves and associated hypersonic devices has turned into a substantial topic in the basic and applied research domain. Thus, graphene represents a perfect ultrathin material in a spatial direction [41].

Combining the graphene with a conventional semiconductor material enables to form a complex multilayer structure. The significant difference in elastic properties between the graphene and the semiconductor layers enables reaching new and unique physical properties associated with this complex structure. This is due especially to the significant difference in density and speed of sound in graphene and conventional semiconductors. Therefore, it can be predicted that the multilayer structure based on graphene-semiconductor can show some interesting features for the modulation and propagation of high-frequency sound waves. The thickness of the graphene layer at the nanoscale is 0.335 nm. Therefore, a graphene-based multilayer structure can be used to design and manufacture hypersonic devices in the THz domain. In addition, such a structure has an advantage for graphene-based phononic crystal engineering with an acoustic band gap at high frequencies [42] to study different elastic properties such as surface and defect modes [34]. Even though the study of graphene in the high frequency sound regime is a growing field with numerous possible applications, few works have been devoted to the study of the mechanical properties of multilayerd structures based on graphene. Zhang et al. [43] studied theoretically the transmission coefficient and band structure of graphene-semiconductor multilayer structures, but only for longitudinal modes at normal incidence. However, the oblique incidence, where longitudinal and transverse waves are coupled, exhibits more interesting results for bulk band structures. In addition, the local and total densities of states (DOS) corresponding to the surface of a SL, and in particular the existence of resonant and localized modes associated with the surface of a graphene-semiconductor SL has not been the subject of any previous study. The theoretical results are illustrated by numerical applications for graphene-Si SLs.

In this paper, we are interested in the elastic properties of graphene-based 1D phononic crystal. In addition to the band structures, we examine the existence and behavior of localized and resonant sagittal modes associated with the free surface of a graphene-semiconductor SL with the help of local and total DOS. These modes are polarized in the sagittal plane, defined by the incident wavevector and the normal to the layers. The main motivation of this study is to show that, despite the low thickness of the graphene layer, the latter when placed periodically in a semiconductor, it allows for opening wide gaps for all values of the wave vector $k_{//}$ (parallel to the interfaces). In addition, we show the possibility of existence of surface modes in a semi-infinite SL that ends either with a graphene layer or with a semiconductor layer. Such modes can be qualified as acoustical Tamm states [44]. Numerical illustrations of the band structures and surface modes are given for graphene-Si SLs, and the surface layer can be either Si or graphene. The advantage of using graphene-Si SL among different bilayer SLs includes the following: (i) the graphene monolayer is nanoscale (with 0.335 nm thickness), which yields band gaps at very high frequency (THz domain) and (ii) a big mismatch between elastic properties of graphene and Si, which gives rise to large gaps.

This paper is organized as follows: in Section 2 and Section 3, we present the method of calculation which is based on the Green’s function for the infinite and semi-infinite SLs, respectively. This approach enables deducing the DOS (Section 4) and therefore dispersion curves of infinite and semi-infinite SLs composed of graphene-semiconductor bilayers. Due to the coupling between longitudinal and transverse waves, the calculation becomes very cumbersome, which is why we preferred to give the explicit expressions of the Green’s functions and DOS in the Supplementary Material. The numerical results obtained in this study are presented and discussed in Section 5 for graphene-Si SLs. The main conclusions are given in Section 6.

## 2. Green’s Function of an Infinite Superlattice

**Figure 1:**
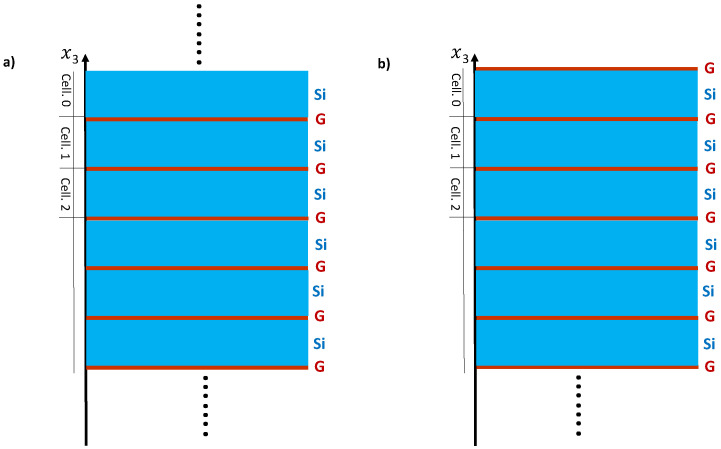
(**a**) Schematic representation of the infinite SL formed of graphene and silicon semiconductor layers denoted G−Si and characterized by the thicknesses $d_{G}$ and $d_{Si}$. The period of the SL is $D=d_{Si}+d_{G}$. The $x_{3}$ axis of the SL is perpendicular to these layers; (**b**) schematic representation of the semi-infinite SL ended by graphene layer. The surface layer can also be of Si type.

Consider a SL composed of two alternating layers labeled i=1 (graphene) and i=2 (semiconductor) (Figure 1a). The calculation is performed by considering the constituents of both layers being of hexagonal symmetry with the c-axis oriented along the axis of the SL in such a way the interfaces are (0001). Each medium is characterized by its density ρ and elastic constants $C_{11},C_{33},C_{13},C_{12},C_{44}$. Due to the isotropy of the (0001) plane in a hexagonal crystal, the $x_{1},x_{2}$ axes can be rotated in such a way that the wave-vector $k_{//}$ parallel to the layers becomes along the $x_{1}$ axis [45]. The above parameters apply for the graphene layer, whereas, in the case of Si, which crystallizes in cubic structure characterized by the elastic constants $C_{11},C_{12}$ and $C_{44}$ we should take in the calculation $C_{33}=C_{11}$ and $C_{13}=C_{12}$ [45]. This geometry enables studying separately pure transverse modes polarized along the $x_{2}$ axis, which become decoupled from sagittal modes polarized in the sagittal plane ($x_{1},x_{3}$). In this work, we are interested in the latter modes involving both transverse and longitudinal waves.

The calculation of the elements of the Green’s function g(D,D) in the whole space *D* of the SL [42] first requires the knowledge of the elements $g(M_{m},M_{m})$ in the restricted space of the $M_{m}$ interfaces, and the index m denotes the i-layer belonging to the cell n: m≡(n,i) and $M_{m}$ represents the space of the two interfaces of layer i: $M_{m}=\left(n,i,\pm \frac{d_{i}}{2}\right),i=1,2$. However, in practice, $g(M_{m},M_{m})$ is obtained by calculating its inverse $g^{-1}\left(M_{m},M_{m}\right)$; it may be simply constructed from a juxtaposition of the matrices $g_{si}^{-1}\left(M_{m},M_{m}\right)$ corresponding to the successive layers i=1 and i=2 [42]. Let us recall that $g_{si}^{-1}\left(M_{m},M_{m}\right)$ can be written as [42]:

(1) $$g_{si}^{-1}\left(M_{m}M_{m}\right)=\left(\begin{array}{cc} A_{i} & \;\;B_{i}\\ B_{i}^{*} & \;\;A_{i}^{*} \end{array}\right),$$

where

(2) $$A_{i}=\left(\begin{array}{cc} a_{i} & \;\;iq_{i}\\ -iq_{i} & \;\;b_{i} \end{array}\right),\;B_{i}=\left(\begin{array}{cc} d_{i} & \;\;if_{i}\\ if_{i} & \;\;e_{i} \end{array}\right)$$

and $A_{i}^{*}$ and $B_{i}^{*}$ are their respective conjugate complexes. $a_{i},b_{i},e_{i},f_{i}$ and $q_{i}$ are real quantities [42] for media i=1 and i=2. The corresponding expressions are given in the Supplementary Material Section S1. They are defined for a given frequency ω and a given wave vector $k_{//}$ parallel to the interfaces. In addition, let us notice that the elements of the Green’s function $g_{si}^{-1}\left(M_{m}M_{m}\right)$ of the layers (Equation (1)) are 2×2 matrices (Equation (2)) because of the two directions of vibrations $x_{1}$ and $x_{3}$ parallel and perpendicular to the interfaces, respectively.

Therefore, the inverse of the Green’s function of an infinite SL $g^{-1}\left(M_{m},M_{m}\right)$ becomes a tridiagonal matrix as follows [42]:

(3) $$g^{-1}\left(M_{m},M_{m}\right)=\left(\begin{array}{cccccccc} \setminus & \;\;\;\;\setminus & \;\;\;\;\setminus & \;\;\;\; & \;\;\;\; & \;\;\;\; & \;\;\;\; & \;\;\;\\ \; & \;\;\;\;\setminus & \;\;\;\;\setminus & \;\;\;\;\setminus & \;\;\;\; & \;\;\;\; & \;\;\;\; & \;\;\;\\ \; & \;\;\;\; & \;\;\;\;B_{1}^{*} & \;\;\;\;A_{1}^{*}+A_{2} & \;\;\;B_{2} & \;\;\;\; & \;\;\;\; & \;\;\;\\ \; & \;\;\;\; & \;\;\;\; & \;\;B_{2}^{*} & \;\;\;A_{2}^{*}+A_{1} & \;B_{1} & \;\;\;\; & \;\;\;\\ \; & \;\;\;\; & \;\;\;\; & \;\;\;\; & \;\;\;B_{1}^{*} & \;\;\;A_{1}^{*}+A_{2} & \;\;\;B_{2} & \;\;\;\\ \; & \;\;\;\; & \;\;\;\; & \;\;\;\; & \;\;\;\; & \;\;\setminus & \;\;\;\setminus & \;\;\;\setminus \\ \; & \;\;\;\; & \;\;\;\; & \;\;\;\; & \;\;\;\; & \;\;\;\; & \;\;\setminus & \;\;\;\setminus \end{array}\right)$$

Taking into account the periodicity $D=d_{1}+d_{2}$ along the direction $x_{3}$ of a SL, the Fourier transformation $g_{c}^{-1}\left(k_{3};M,M\right)$, within a unit cell, leads to the next reduced matrix:

(4) $$g_{c}^{-1}\left(k_{3};M,M\right)=\left(\begin{array}{cc} A_{1}+A_{2}^{*} & \;\;B_{1}+B_{2}^{*}e^{-ik_{3}D}\\ B_{1}^{*}+B_{2}e^{ik_{3}D} & \;\;A_{1}^{*}+A_{2} \end{array}\right),$$

Here, $k_{3}$ is the Bloch wave vector along the axis of the SL and can be considered inside the first Brillouin zone (BZ) $-\pi /D<k_{3}<\pi /D$. Replacing $A_{1},A_{2},B_{1},B_{2}$ and their complex conjugates by their corresponding values (Equation (2)), $g_{c}^{-1}\left(k_{3};M,M\right)$ can be written as

(5) $$g_{c}^{-1}\left(k_{3};M,M\right)=\left[\begin{array}{cccc} a_{1}+a_{2} & \;\;i(q_{1}-q_{2}) & \;\;d_{1}+d_{2}e^{-ik_{3}D} & \;\;i(f_{1}-f_{2}e^{-ik_{3}D})\\ -i(q_{1}-q_{2}) & \;\;b_{1}+b_{2} & \;\;i(f_{1}-f_{2}e^{-ik_{3}D}) & \;\;e_{1}+e_{2}e^{-ik_{3}D}\\ d_{1}+d_{2}e^{ik_{3}D} & \;\;-i(f_{1}-f_{2}e^{ik_{3}D}) & \;\;a_{1}+a_{2} & \;\;-i(q_{1}-q_{2})\\ -i(f_{1}-f_{2}e^{-ik_{3}D}) & \;\;e_{1}+e_{2}e^{ik_{3}D} & \;\;i(q_{1}-q_{2}) & \;\;b_{1}+b_{2} \end{array}\right]$$

The dispersion relation is obtained from the equation $\det(g_{c}^{-1}\left(k_{3};M,M\right))=0$ [42], namely

(6) $$\alpha_{0}\left[\cos^{2}\left(k_{3}D\right)-2\delta \cos\left(k_{3}D\right)+\gamma \right]=0$$

where $\alpha_{0}$, δ and γ are real parameters functions of the terms $a_{i},b_{i},e_{i},f_{i}$ and $q_{i}$ for i=1,2. They are functions of $k_{//}$ and ω together with the material properties of the structure (see the Supplementary Material Section S1). Thus, we obtain two solutions for $\cos(k_{3}D)$ which can be written as follows:

(7) $$\eta_{1}=\delta +{\left(\delta^{2}-\gamma \right)}^{1/2}$$

and

(8) $$\eta_{2}=\delta -{\left(\delta^{2}-\gamma \right)}^{1/2}$$

From $\eta_{1}$ and $\eta_{2}$, we deduce the corresponding expressions of $t_{1}$ and $t_{2}$ using the equation

(9) $$t_{i}=\left\{\begin{array}{c} \eta_{i}+{\left(\eta_{i}^{2}-1\right)}^{1/2},\;\;\;\;\eta_{i}<-1,\\ \eta_{i}\pm i{\left(1-\eta_{i}^{2}\right)}^{1/2},\;\;\;\;-1<\eta_{i}<+1,\\ \eta_{i}-{\left(\eta_{i}^{2}-1\right)}^{1/2},\;\;\;\;\eta_{i}>1. \end{array}\right.$$

Let us recall that each of these parameters labeled “*t*” is associated with a wave vector $k_{3}$ in the direction $x_{3}$ such that

(10) $$t=e^{ik_{3}D}.$$

However, if $k_{3}$ is a solution of Equation (6), $-k_{3}$ or (1/t) is also a solution. Consequently, the two pairs of solutions of $k_{3}$ associated respectively with $t_{1}$ and $t_{2}$, for a given $k_{//}$ and ω can be written as

(11) $$K_{1}+iL_{1},\;\;-\left(K_{1}+iL_{1}\right),\;\;\left(K_{2}+iL_{2}\right),\;\;-\left(K_{2}+iL_{2}\right).$$

Depending on whether $k_{3}$ is purely real or contains an imaginary part, the corresponding wave can propagate (i.e., band) or not (i.e., gap) in the SL. We can distinguish the following two cases (for ω and $k_{//}$ fixed) depending on whether ${\left(\delta^{2}-\gamma \right)}^{1/2}$ (Equations (7) and (8)) is real or imaginary:

(**i**) ${\left(\delta^{2}-\gamma \right)}^{1/2}$ real; then $\eta_{1}$ or $\eta_{2}$ are also real. Moreover, if $|\eta_{1}\left|\leq 1(\right|t_{1}\left|=1\right)$ or $|\eta_{2}\left|\leq 1(\right|t_{2}\left|=1\right)$, the corresponding $k_{3}$ is real ($L_{1}=0$ or $L_{2}=0$) and the frequency belongs to a SL band; however, if both $|\eta_{1}|>1$ and $|\eta_{2}|>1$, the $k_{3}$ are either purely imaginary $\left(K_{1}=K_{2}=0\right)$ or complex but with $K_{1}=K_{2}=\frac{\pi }{D}$ and the frequency is within a SL gap.

(**ii**) ${\left(\delta^{2}-\gamma \right)}^{1/2}$ imaginary; then, $\eta_{1}$ and $\eta_{2}$ are conjugates complex and the corresponding wave vectors $k_{3}$ are written in the form:

(12) K+iL,−(K+iL),(K−iL),−(K−iL),

these frequencies correspond to the SL gaps. The set of possible behaviors for the wave vectors $k_{3}$ will be discussed later in Section 5.

By inverting the matrix $g^{-1}\left(k_{3};M,M\right)$ given by the Equation (5), the 16 elements of the response function in the interface space (in reciprocal space) may formally be written in the form:

(13) $$g_{\alpha \beta }\left(k_{3};\upsilon ,\upsilon^{\prime },\mu \right)=\frac{1}{\alpha_{0}\left[\cos\left(k_{3}D\right)-\eta_{1}\right]\left[\cos\left(k_{3}D\right)-\eta_{2}\right]}\sum_{\mu =-2}^{\mu =2}R_{\alpha \beta }\left(\upsilon ,\upsilon^{\prime },\mu \right)e^{i\mu k_{3}D}$$

where α and β represent the directions of vibration $x_{1},x_{3}$ also noted 1,3$(\alpha ,\beta =x_{1},x_{3}$ or 1,3), υ and $\upsilon^{\prime }$ indicate the positions of the interfaces of the layer i=1 of the SL $\left(\upsilon ,\upsilon^{\prime }=-\frac{d_{1}}{2},\frac{d_{1}}{2}\right)$. $R_{\alpha \beta }\left(\upsilon ,\upsilon^{\prime },\mu \right)\left(\alpha ,\beta =1,3\right)$ are functions of the parameters $a_{i},b_{i},d_{i},e_{i},f_{i}$ and $q_{i}$ of the layers i=1 and i=2 which constitute the SL. $\eta_{1}$ and $\eta_{2}$ are defined by Equations (7) and (8).

By using the reciprocal Fourier transform and the residue theorem, the elements of the Green’s function in the space of interfaces (in real space) can be written explicitly in the form:

(14) $$g_{\alpha \beta }\left(n,i=1,\upsilon ;n^{\prime },i=1,\upsilon^{\prime }\right)=\frac{1}{\alpha_{0}\left(\eta_{1}-\eta_{2}\right)}\sum_{\mu =-2}^{\mu =2}R_{\alpha \beta }\left(\upsilon ,\upsilon^{\prime },\mu \right)\left[\frac{t_{1}^{|n-n^{\prime }+\mu |+1}}{t_{1}^{2}-1}-\frac{t_{2}^{|n-n^{\prime }+\mu |+1}}{t_{2}^{2}-1}\right]$$

$t_{1}$ and $t_{2}$ are obtained from $\eta_{1}$ and $\eta_{2}$ using Equation (9). The explicit expressions of the Green’s functions $g_{\alpha \beta }\left(n,i=1,\upsilon ;n^{\prime },i=1,\upsilon^{\prime }\right)$ in the space of interfaces are given in the Supplementary Material Section S2.

The Green’s function between any two points in the infinite SL is obtained from the following expression [42]:

(15) $$\begin{array}{c} g\left(n,i,x_{3};n^{\prime },i^{\prime },x_{3}^{\prime }\right)=\delta_{nn^{\prime }}\delta_{ii^{\prime }}\left[G_{i}\left(x_{3},x_{3}^{\prime }\right)-G_{i}\left(x_{3},M_{m}\right)G_{i}^{-1}\left(M_{m},M_{m^{\prime }}\right)G_{i}\left(M_{m},x_{3}^{\prime }\right)\right]+\\ \;\;\;\;\;\;\;\;G_{i}\left(x_{3},M_{m^{\prime }}\right)G^{-1}\left(M_{m},M_{m}\right)g\left(M_{m},M_{m^{\prime }}\right)G_{i}^{-1}\left(M_{m^{\prime }},M_{m^{\prime }}\right)G_{i}\left(M_{m^{\prime }},x_{3}^{\prime }\right), \end{array}$$

where $g(M_{m}M_{m}^{\prime })$ is a 4×4 matrix whose elements are given by Equation (14) with $M_{m}\equiv \left(n,i,\pm \frac{d_{i}}{2}\right)$ and $M_{m}^{\prime }\equiv \left(n^{\prime },i^{\prime },\pm \frac{d_{i}^{\prime }}{2}\right)$. $G_{i}\left(x_{3},x_{3}^{\prime }\right),G_{i}^{-1}\left(M_{m},M_{m}\right),G_{i}\left(x_{3},M_{m}\right),$ and $G_{i}\left(M_{m}^{\prime },x_{3}^{\prime }\right)$ are (2×2), (4×4), (2×4) and (4×2) matrices, respectively, whose expressions are given in the Supplementary Material Section S3.

## 3. Green’s Function of a Semi-Infinite Superlattice

Let us consider the semi-infinite SL located in the negative half-space $x_{3}<0$ and terminated by a complete layer of type i=1 (Figure 1b). This SL is created from the infinite one by removing for example the layer (n=0,i=2). The perturbation operator that allows such operation to be performed is given by

(16) $$V\left(M_{1}M_{1}\right)=-\left(\begin{array}{cc} A_{2} & \;\;B_{2}\\ B_{2}^{*} & \;\;A_{2}^{*} \end{array}\right).$$

$A_{2}$ and $B_{2}$ are defined from Equation (2) and $M_{1}$ represents the space of the interfaces affected by the perturbation V, namely $M_{1}\equiv \left\{\left(n=0,i=1,\frac{d_{1}}{2}\right),\left(n=1,i=1,-\frac{d_{1}}{2}\right)\right\}$. By taking into account the two degrees of vibration, four states are affected in such operation.

From the elements of the bulk Green’s function (Equation (14)) and those of the perturbation operator (Equation 16), one can deduce the explicit expressions of the surface response operator (see the Supplementary Material Section S4), namely [42]

(17) $$A\left(M_{0},M_{m}\right)=\sum_{M_{1}}V\left(M_{0},M_{1}\right)g\left(M_{1},M_{m}\right),$$

where $M_{0}$ represents the surface of the semi-infinite SL (Figure 1b): $M_{0}\equiv \left(n=0,i=1,\frac{d_{1}}{2}\right)$ and $M_{m}$ is the interface space of the semi-infinite SL: $M_{m}\equiv \left(n,i=1,\pm \frac{d_{1}}{2}\right)$.

The elements of the response function in the interface space of the semi-infinite SL can be written as [42]

(18) $$\begin{array}{c} d\left(n,1,\pm \frac{d_{1}}{2};n^{\prime },1,\pm \frac{d_{1}}{2}\right)=g\left(n,1,\pm \frac{d_{1}}{2};n^{\prime },1,\pm \frac{d_{1}}{2}\right)-\\ \;\;\;\;\;\;\;\;g\left(n,1,\pm \frac{d_{1}}{2};0,1,\frac{d_{1}}{2}\right)\Delta^{-1}\left(0,1,\frac{d_{1}}{2};0,1,\frac{d_{1}}{2}\right)A\left(0,1,\frac{d_{1}}{2};n^{\prime },1,\pm \frac{d_{1}}{2}\right), \end{array}$$

where the operator [42] $\Delta (0,1,\frac{d_{1}}{2};0,1,\frac{d_{1}}{2})$ is given by [42]

(19) $$\Delta \left(0,1,\frac{d_{1}}{2};0,1,\frac{d_{1}}{2}\right)=I+A\left(0,1,\frac{d_{1}}{2};0,1,\frac{d_{1}}{2}\right)$$

The elements of $g(n,1,\pm \frac{d_{1}}{2};n^{\prime },1,\frac{d_{1}}{2})$ are given in the Supplementary Material Section S2, while the explicit expressions of the elements of the operators $d(n,1,\pm \frac{d_{1}}{2};n^{\prime },1,\pm \frac{d_{1}}{2})$ and $\Delta (0,1,\frac{d_{1}}{2};0,1,\frac{d_{1}}{2})$ are given in the Supplementary Material Section S5.

Finally, the expression of the Green’s function between any two points of the semi-infinite SL is obtained in the same way as in Equation (15) by replacing the matrix $g(M_{m}M_{m}^{\prime })$ (Equation (14)) by $d(M_{m}M_{m}^{\prime })$ (Equation (18)), namely

(20) $$\begin{array}{c} d\left(n,i,x_{3};n^{\prime },i^{\prime },x_{3}^{\prime }\right)=\delta_{nn^{\prime }}\delta_{ii^{\prime }}\left[G_{i}\left(x_{3},x_{3}^{\prime }\right)-G_{i}\left(x_{3},M_{m}\right)G_{i}^{-1}\left(M_{m},M_{m^{\prime }}\right)G_{i}\left(M_{m},x_{3}^{\prime }\right)\right]+\\ \;\;\;\;\;\;\;\;G_{i}\left(x_{3},M_{m^{\prime }}\right)G^{-1}\left(M_{m},M_{m}\right)d\left(M_{m},M_{m^{\prime }}\right)G_{i}^{-1}\left(M_{m^{\prime }},M_{m^{\prime }}\right)G_{i}\left(M_{m^{\prime }},x_{3}^{\prime }\right) \end{array}$$

## 4. Densities of States

Knowing the response functions given in the Supplementary Material Section S5, we obtain for a given value of $k_{//}$, the local and total densities of states for a semi-infinite SL.

### 4.1. Local Density of States

The local density of states on the plane $\left(n,i,x_{3}\right)$ is given by

(21) $$n_{\alpha }\left(\omega^{2},k_{//};n,i,x_{3}\right)=-\frac{1}{\pi }Imd_{\alpha \alpha }^{+}\left(\omega^{2},k_{//}|n,i,x_{3};n,i,x_{3}\right)\;\;\;\;\left(\alpha =1,3\right)$$

where $d^{+}\left(\omega^{2}\right)=\lim_{\epsilon \to 0}d\left(\omega^{2}+i\epsilon \right)$ and $d(\omega^{2})$ is the response function whose elements are given in the Supplementary Material S6.

### 4.2. Total Density of States

For a given value of the wavevector $k_{//}$, the total DOS is obtained by integrating over $x_{3}$ the local DOS and summing over n, i and α. In particular, we are interested in calculating the difference between the DOS of the semi-infinite SL and that of an infinite SL with the same number of layers as the semi-infinite SL. This variation $\Delta n(\omega^{2})$ can be written as the sum of the variations of DOS for layers 1 and 2, as

(22) $$\Delta n\left(\omega^{2}\right)=\Delta_{1}n\left(\omega^{2}\right)+\Delta_{2}n\left(\omega^{2}\right)$$

where

(23) $$\Delta_{1}n\left(\omega^{2}\right)=-\frac{\rho_{1}}{\pi }Imtr\sum_{n=-\infty }^{0}\int_{-\frac{d_{1}}{2}}^{\frac{d_{1}}{2}}\left[d\left(n,i=1,x_{3};n,i=1,x_{3}\right)-g\left(n,i=1,x_{3};n,i=1,x_{3}\right)\right]dx_{3}$$

and

(24) $$\Delta_{2}n\left(\omega^{2}\right)=-\frac{\rho_{2}}{\pi }Imtr\sum_{n=-\infty }^{-1}\int_{-\frac{d_{2}}{2}}^{\frac{d_{2}}{2}}\left[d\left(n,i=2,x_{3};n,i=2,x_{3}\right)-g\left(n,i=2,x_{3};n,i=2,x_{3}\right)\right]dx_{3}$$

d and g are the Green’s functions of semi-infinite and infinite SLs, respectively. From Equations (15), (20), (23) and (24), we obtain

(25) $$\Delta_{1}n\left(\omega^{2}\right)=-\frac{\rho_{1}}{\pi }Im\left(tr\int_{-\frac{d_{1}}{2}}^{\frac{d_{1}}{2}}G_{1}\left(x_{3},M_{m}\right)T\left(M_{m},M_{m}\right)G_{1}\left(M_{m},x_{3}\right)dx_{3}\right)$$

where

(26) $$T\left(M_{m},M_{m}\right)=G_{1}^{-1}\left(M_{m},M_{m}\right)\sum_{n=-\infty }^{0}\left[d\left(M_{m},M_{m}\right)-g\left(M_{m},M_{m}\right)\right]G_{1}^{-1}\left(M_{m},M_{m}\right),$$

and

(27) $$\Delta_{2}n\left(\omega^{2}\right)=-\frac{\rho_{2}}{\pi }Im\left(tr\int_{-\frac{d_{2}}{2}}^{\frac{d_{2}}{2}}G_{2}\left(x_{3},M_{m}\right)T^{\prime }\left(M_{m},M_{m}\right)G_{2}\left(M_{m},x_{3}\right)dx_{3}\right)$$

where

(28) $$T^{\prime }\left(M_{m},M_{m}\right)=G_{2}^{-1}\left(M_{m},M_{m}\right)\sum_{n=-\infty }^{0}\left[d\left(M_{m},M_{m}\right)-g\left(M_{m},M_{m}\right)\right]G_{2}^{-1}\left(M_{m},M_{m}\right),$$

$M_{m}$ denotes the space of the interfaces $\left(n,i,\pm \frac{d_{1}}{2}\right)$.

The details of calculation of the quantities $\Delta_{1}n\left(\omega^{2}\right)$ and $\Delta_{2}n\left(\omega^{2}\right)$ are given in the Supplementary Material Section S6. The summation over n in Equations (26) and (28) can be easily performed since the Green’s functions between brackets in these equations are composed of geometric series (see the Supplementary Material Section S6). Similarly, in Equations (25) and (27), the integration over $x_{3}$ can be easily performed since the elements of Green’s functions are composed only of exponential terms (see the Supplementary Material Section S6). Finally, the trace in Equations (25) and (27) is taken over the components 11 and 33 corresponding to the contribution of the sagittal modes considered in this work.

## 5. Numerical Calculations of Dispersion Curves, Surface Modes, and Densities of States

In this section, we consider a periodic structure composed of layers based on silicon and graphene. The thickness of the silicon layer is $d_{Si}=11$ nm and that of the graphene layer is $d_{G}=0.335$ nm, the period of the SL being $D=d_{Si}+d_{G}$. The elastic parameters used in this calculation are given in Table 1 [46]. In this work, we show the possibility of existence of large forbidden bands as well as localized and resonant surface modes.

As previously reported in Section 2, there are two pairs of $k_{3}$ (Equation (11)) for each value of $k_{//}$ and ω. Each pair of $k_{3}$ (the first for example) can take four different forms, namely

(29) $$\left(i\right)\;\mathrm{pure}\;\mathrm{real}\;\;\left(L_{1}=0\right),$$

(30) $$\left(ii\right)\;\mathrm{pure}\;\mathrm{imaginary}\;\;\left(K_{1}=0\right),$$

(31) $$\left(iii\right)\;\mathrm{complex},\;\mathrm{but}\;\mathrm{with}\;\;K_{1}=\pm \pi /D,$$

(32) $$\left(iv\right)\;\mathrm{complex},\;\mathrm{with}\;\;\;K_{1}\neq \pm \pi /D.$$

However, in case (iv), the two pairs of $k_{3}$ are given by Equation (12).

**Figure 2:**
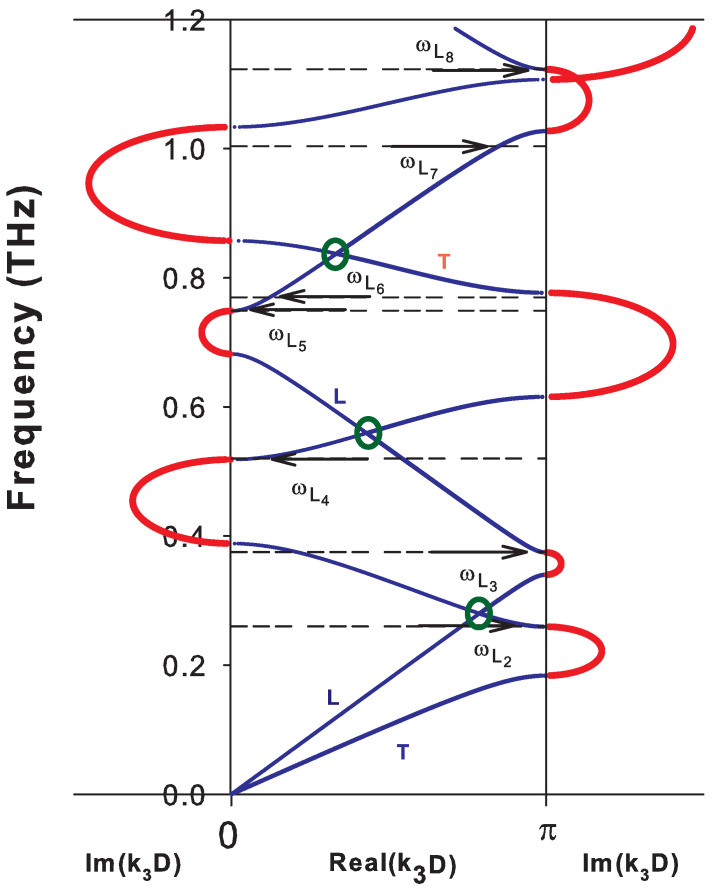
Complex band structure of sagittal modes in an Si-graphene based SL $d_{Si}=11$ nm, $d_{G}=0.335$ nm, $D=d_{Si}+d_{G}$ and $k_{//}D=0$. The blue curves correspond to real $k_{3}$ inside the Brillouin zone (between 0 and π/D). The red curves at the left part of the figure correspond to a purely imaginary $k_{3}$, whereas red curves on the right part of the figure correspond to a complex $k_{3}$ when its real part is equal to π/D. L and T refer to pure longitudinal and pure transverse modes, respectively. The horizontal arrows indicate the positions of the frequencies of surface localized modes labeled $L_{i}$.

**Figure 3:**
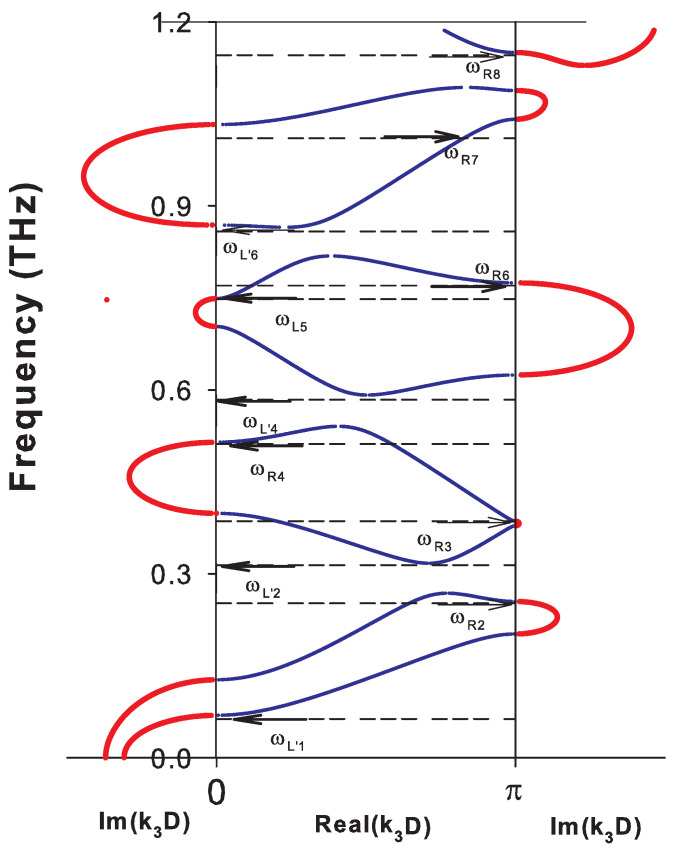
Same as in Figure 2, but for $k_{//}D=1$. Blue and red curves correspond to real and imaginary parts of the wavevector $k_{3}$ inside the Brillouin zone, respectively. The horizontal arrows indicate the frequency positions of the surface localized $\left(L_{i}\right)$ and resonant $\left(R_{i}\right)$ modes.

To give a better insight about all these cases, we have represented the complex band structure for $k_{//}D=0$, (Figure 2) and $k_{//}D=1$, (Figure 3). In Figure 2$\left(k_{//}D=0\right)$, the modes have either a purely transverse (T) or purely longitudinal (L) polarization and propagate independently of each other. Because of the difference in sound velocities of transverse (T) and longitudinal (L) phonons, the dispersion curves intersect at a number of points within the BZ indicated by green circles. For each polarization, there are direct gaps at the center and at the limit of the BZ of the periodic structure of the SL (Figure 2). The introduction of a coupling between the waves of different polarizations (see Figure 3 for $k_{//}D=1$) gives rise, in addition to direct gaps at the edges of the BZ, to indirect gaps within the BZ at the anti-crossing points. These anti-crossings occur in a region where two dispersion curves meet. When $k_{//}D$ increases, the indirect gaps (anti-crossings) become wider and may even turn into a direct gap for some values of $k_{//}D$. Note that the imaginary parts of the wave vector $k_{3}$ in Figure 2 and Figure 3 give the attenuation of localized and resonant (or semi-localized) waves in the minigaps and minibands, respectively, as will be shown below.

**Figure 4:**
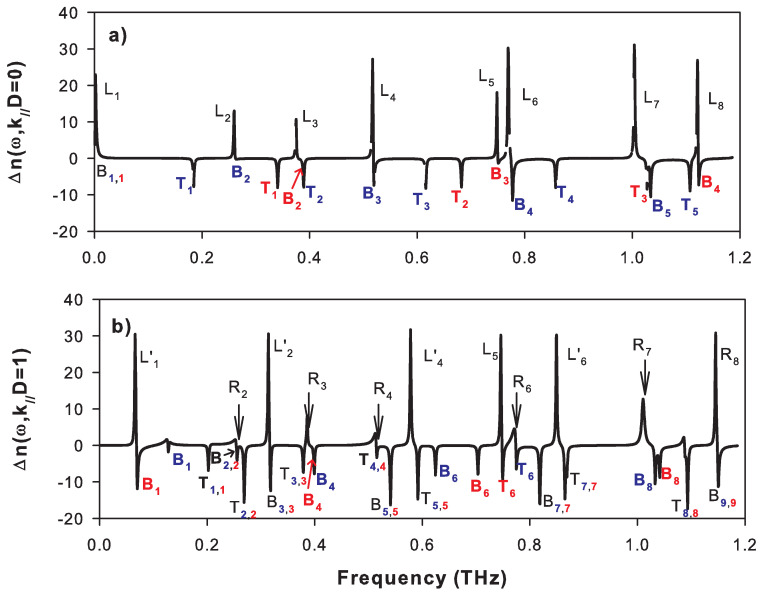
Variation of the density of states between the semi-infinite SL terminated by a graphene layer of thickness $d_{G}=0.335$ nm and the same volume of an infinite SL (Equations (23) and (24)) versus the frequency (in THz), for $k_{//}D=0$ (**a**) and $k_{//}D=1$ (**b**). $L_{i}$ and $R_{i}$ refer to localized and resonant modes, respectively. $B_{i}$ and $T_{i}$ indicate the bottom and top of the two types of bands.

The creation of the free surface of the SL gives rise to localized and resonant modes inside the SL gaps. The frequencies of these modes are indicated by arrows in Figure 2 and Figure 3 when the SL is ended by a graphene layer at the surface. These modes are called localized when the two corresponding wave vectors are complex (see for example the modes $\omega_{L_{1}},\omega_{L_{2}^{\prime }},\omega_{L_{1}^{\prime }},\omega_{L_{4}}$ and $\omega_{L_{5}^{\prime }}$ in Figure 3) and semi-localized (or resonant) when one of the wave vectors is complex and the other is real (see, for example, the modes $\omega_{R_{2}},\omega_{R_{3}},\omega_{R_{5}}$ and $\omega_{R_{6}}$ in Figure 3). These different surface modes are obtained from the maxima of the peaks in the DOS illustrated in Figure 4a,b for $k_{//}D=0$ and $k_{//}D=1$, respectively. The δ functions that appear in these figures are enlarged by adding a small imaginary part ϵ to the frequency ω.

**Figure 5:**
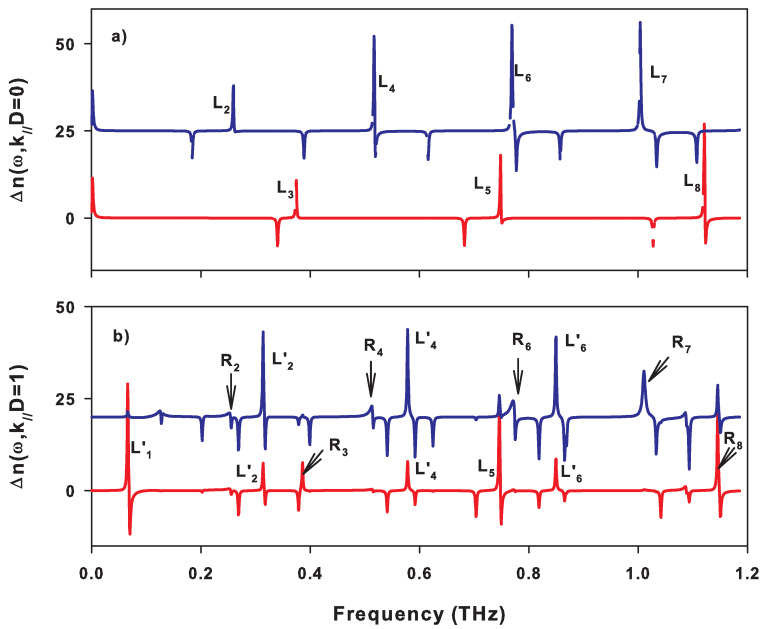
Same as in Figure 4, where the longitudinal (blue curves) and transverse (red curves) components are separated in the DOS, for $k_{//}D=0$ (**a**) and $k_{//}D=1$ (**b**).

In Figure 4a, we have chosen $k_{//}D=0$ and therefore the sagittal modes are split into purely longitudinal and purely transverse modes as shown in Figure 5a where we separated the longitudinal (blue curves) and transverse (red curves) contributions in the variation of DOS. In Figure 4a, the peaks $L_{2},L_{4},L_{6}$ and $L_{7}$ are localized surface modes of longitudinal polarization, while the peaks $L_{3},L_{5}$ and $L_{8}$ correspond to surface modes of transverse polarization, as shown in Figure 5a. Figure 4a and Figure 5a also show δ peaks of weight −1/4 (anti-resonances) at the boundaries of the bands where $Re(k_{3}D)=0$ and $Re(k_{3}D)=\pi$ (see Figure 2). These peaks are denoted $B_{i}$ and $T_{i}$ and they are associated, respectively, with the bottom and the top of the bands i given by the two wave vectors $K_{1}+iL_{1}$ and $K_{2}+iL_{2}$ such as $L_{1}=0$ or $L_{2}=0$ [47,48]. These two types of band structures will be illustrated in detail on the projected band structures presented later, where we reported the frequency as a function of the wave vector $k_{//}$.

When $k_{//}D$ becomes different from zero, the longitudinal and transverse modes become coupled, and some of the localized modes at $k_{//}D=0$ may now fall within the bands of the SL. Such modes may radiate their energy within the bands and become, therefore, resonant modes (also known as leaky waves). This is the case, for example, for localized modes at $k_{//}D=0$ denoted $L_{2},L_{4},L_{6},L_{7}$ in Figure 4a which become resonant modes at $k_{//}D=1$, denoted respectively $R_{2},R_{4},R_{6},R_{7}$ in Figure 4b. The intensities of these resonances decrease as $k_{//}D$ increases. In addition, we observe the opening of new indirect gaps at $k_{//}D=1$ in the frequency ranges 0.27–0.31 THz, 0.54–0.58 THz, and 0.81–0.86 THz (see Figure 3). In these gaps, new localized modes labeled $L_{2}^{\prime },L_{4}^{\prime }$ and $L_{6}^{\prime }$ appear in Figure 3 and Figure 4b. We can also note the existence of δ peaks of weight (−1/4) at the limits of the bands. These peaks do not seem to have exactly the same weight because of divergences in ${\left(\omega -\omega_{Bi}\right)}^{-1/2}$ or ${\left(\omega -\omega_{Ti}\right)}^{-1/2}$ ($\omega_{Bi}$ and $\omega_{Ti}$ being the frequencies of the bottom and top of each allowed band of SL) existing at the boundaries of the bands in the DOS of the one-dimensional systems. However, the localized modes $L_{2}^{\prime },L_{4}^{\prime },L_{6}^{\prime }$ which fall inside the indirect gaps present a mixture of transverse and longitudinal character.

**Figure 6:**
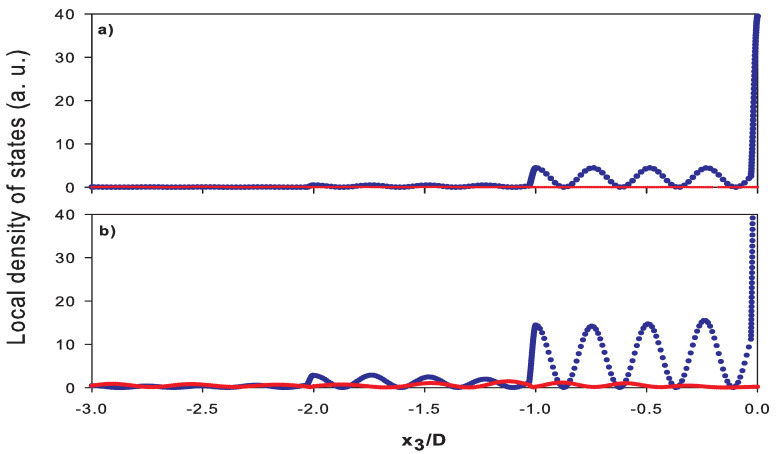
Spatial representation of local density of states for [$k_{//}D=0$, f=1.0025 THz] (**a**) and [$k_{//}D=1$, f=1.0102 THz] (**b**). Blue and red curves correspond to longitudinal and transverse components of the waves, respectively.

An analysis of the local DOS as a function of the space position $x_{3}$ (Figure 6) clearly shows the localization of the different surface modes belonging to different frequency regions. The local DOS reflects the spatial behavior of the square modulus of the displacement field. Figure 6a corresponds to the mode labeled $L_{7}$ (f=1.0025 THz) in Figure 4a and Figure 5a, the longitudinal component in local DOS (blue curve) decreases rapidly from the surface of the SL located at $x_{3}=0$ towards the bulk, while the transverse component (red curve) is equal to zero. Figure 6b corresponds to the mode labeled $R_{7}$ (f=1.0102 THz in Figure 4b and Figure 5b for $k_{//}D=1$, showing a localization of the longitudinal component (blue curve), while the small transverse component (red curve) exhibits an oscillatory behavior in the bulk of the SL with a very low attenuation. One can see that the longitudinal component still remains very localized at the surface even though the surface mode is semi-localized.

As mentioned above, the difference between the spatial localization of the localized and semi-localized (or resonant) modes lies in the fact that, for the first, the two corresponding wave vectors are complex, while, for the latter, one of the wave vectors is complex while the other is real. This situation, which can only occur when the vibrations have at least two degrees of freedom, is without analogue in the case of pure transverse or pure longitudinal modes.

**Figure 7:**
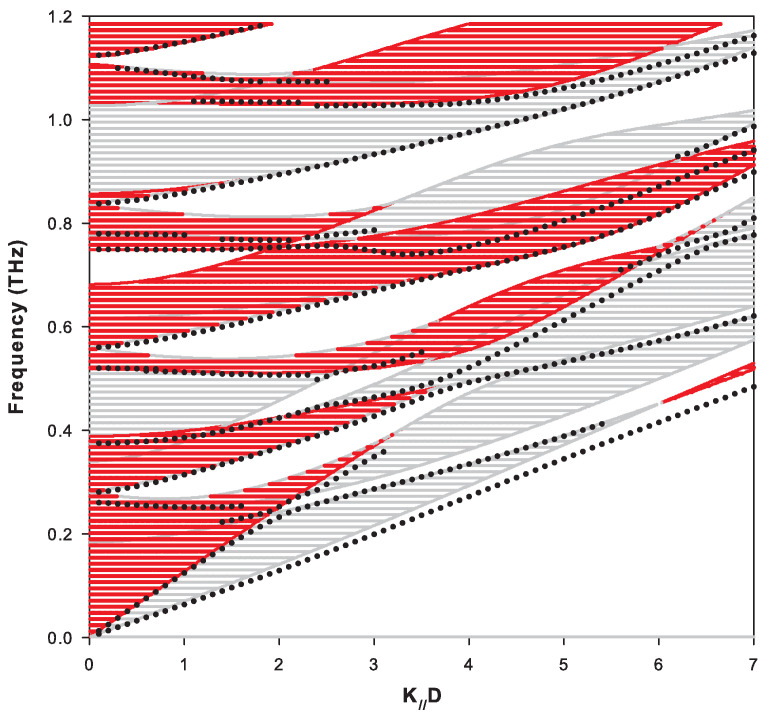
Dispersion of bulk bands and surface modes of an Si-graphene SL terminated by an Si layer of thickness $d_{Si}=11$ nm. The hatched areas with red and gray lines correspond to the bulk bands given by the two polarizations of the waves. Localized and resonant surface modes are represented by full circles.

In Figure 7, we have shown the projected band structure of the bulk and surface modes (i.e., the frequency in THz versus $k_{//}$). The bands associated with each polarization (i.e., $L_{1}=0$ or $L_{2}=0$ in Equation (11)) are represented by red and gray colors. One can distinguish: (i) the frequency regions belonging simultaneously to these two bands, (ii) the frequency regions belonging only to one of the two bands, and (iii) the frequency regions separating these different shaded areas which correspond to the gaps. From the peaks maxima of the density of states associated with localized and resonant surface modes (Figure 4), we have reported the frequencies associated with these maxima by solid circles in Figure 7 for each value of $k_{//}$. We can distinguish localized modes that fall within the minigaps and resonant modes (semi-localized) that fall inside one of the two bands. It may be noted that, apart from the surface mode branch which falls below all the bands, the other branches fall at the vicinity or inside the allowed bands.

**Figure 8:**
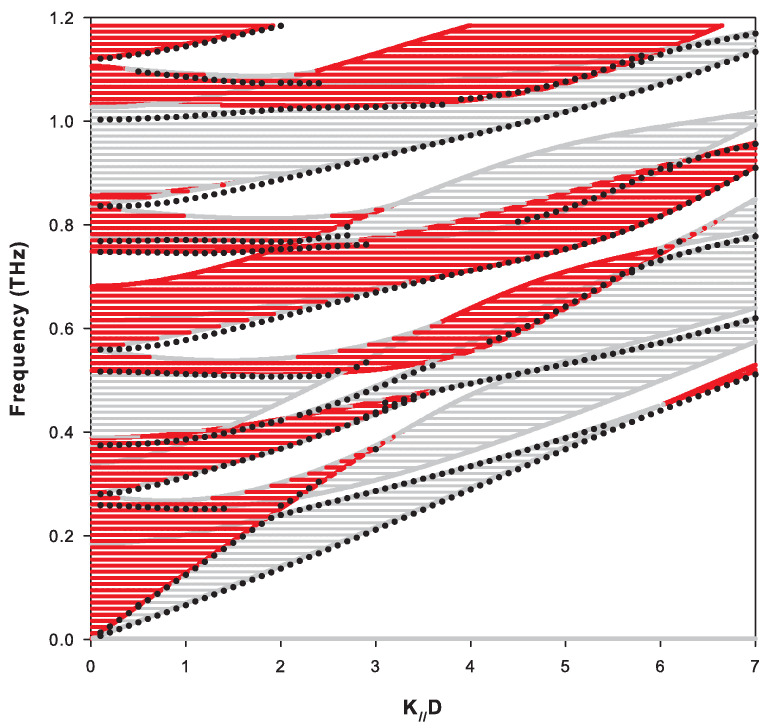
Same as in Figure 7, for the complementary semi-infinite superlattice Si-graphene terminated by a graphene layer of thickness $d_{G}=0.335$ nm at the surface.

Localized and resonant modes depend on the nature of the layer on the surface of the SL. Figure 8 shows the projected band structure of the Si-graphene complementary semi-infinite SL terminated by a graphene layer on the surface. We can notice in this figure the existence of two branches of surface modes that fall in the gaps around f=0.6 THz and f=0.85 THz for $k_{//}D<2$. The other surface modes branches fall inside the bands as resonances.

An interesting result is obtained when considering together the two complementary semi-infinite SLs. Indeed, we have checked numerically that the variation in the DOS is equal to zero for frequencies belonging simultaneously to the two types of bulk bands (hatched areas with both red and gray in Figure 7 and Figure 8). This result is associated with the existence of anti-resonances of weight (−1/2) at the limits of each bulk band of the two complementary SLs (i.e., the loss of one mode per band), and the necessary conservation of the total number of modes shows that it is necessary that surface modes appear to compensate for the loss of these modes. These modes are distributed differently on the two complementary semi-infinite SLs as illustrated in Figure 7 and Figure 8. Despite their apparent similarities, a closer look to these two figures reveals several differences between them as concerns the surface modes. For example, in Figure 7, the lowest surface branch deviates significantly from the bottom edge of the bulk band when $k_{//}D>2$; in contrast, in Figure 8, it always remains very close to the bulk band. In addition, several differences can be observed between the two figures in the details of the resonant modes for $k_{//}D>3$ in the frequency range from 0.4 to 0.9 THz.

## 6. Conclusions

The results presented in this paper are based on a detailed calculation of the Green’s function acoustic waves of sagittal polarization in infinite and semi-infinite SLs composed of graphene-semiconductor bi-layers. These complete Green’s functions can be used to study any physical properties of a semi-infinite SL. This includes the calculations of local and total density of state and determining the dispersion relations of the bulk and surface waves in these structures. The approach of the Green’s functions used in these analyzes can be also used to obtain the displacement field associated with multiple reflections and transmission at different interfaces, even if we have not stressed this point in this work. These quantities can be used, for example, for the study of transmission and reflection coefficients as well as light scattering by acoustic phonons [38,39,49,50].

The density of states allowed us to deduce the dispersion of localized and resonant modes (also called pseudo-modes or leaky waves) of sagittal polarization in the graphene-semiconductor semi-infinite SLs. Due to the coupling of two directions of vibrations, there are two bulk bands corresponding to two polarizations of the waves. The creation of the free surface of the SL gives rise to δ peaks of weight (−1/4) at the limits of each bulk band (or δ peaks of weight (−1/2) if one considers two complementary SLs). Because of the conservation of the total number of states, there should be the appearance of surface localized modes for which the two components of the displacement field (or equivalently the local density of states) are attenuated in the SL as well as semi-localized modes (or pseudo-modes) for which one component is attenuated while the other component propagates in the SL. The advantage of graphene-based phononic crystal lies in the fact that the width of graphene is nanoscale and its elastic properties are much higher than those of Si, which enables getting large gaps in the THz domain.

The surface acoustic modes can be used to realize liquid or bio-sensors when the SL is deposited on a substrate from which an incident wave is launched, while the free surface is in contact with the sample to be detected. Indeed, the surface modes are very sensitive to any nano-object in contact with the surface of the SL, which modifies its elastic properties leading to a noticeable shift of the surface mode frequency like in the Kretschmann configuration using surface plasmon resonance. In addition, this structure can be designed as a plasmonic sensor operating in the optical domain [28,51]. This work is in progress. Finally, it is worth noticing that other interesting physical phenomena can be expected in this structure that go beyond the objectives of the present manuscript. For instance, a lattice dynamical study of the structure would allow for accessing the optical phonons besides the acoustic phonons studied here in the frame of the elasticity theory. In addition, due to the presence of the Dirac point in graphene which behaves as the Fermi surface, the electron–phonon coupling effect similar to Kohn anaomaly in the phonon dispersion curves [52,53] can take place. For instance, let us mention the observation of this phenomenon in the optical [54,55] and acoustical [56] dispersion curves of graphene under strain. In addition, Kohn anomalies have been measured in Nitride-based 6 nm-6 nm metal-semiconductor (HfN-ScN) SL using inelastic x-ray scattering [57].

## Figures and Tables

**Table 1:** Elastic parameters of Si and Graphene.

	$C_{11}\left(\mathrm{GPa}\right)$	$C_{12}\left(\mathrm{GPa}\right)$	$C_{13}\left(\mathrm{GPa}\right)$	$C_{33}\left(\mathrm{GPa}\right)$	$C_{44}\left(\mathrm{GPa}\right)$	$\rho ({\mathrm{Kg}/m}^{3})$
Si	165.77	63.93	63.93	165.77	79.62	2330
Graphene	1109	139	0	38.7	5	1940

## Supplementary Materials

The following are available online at https://www.mdpi.com/2079-4991/10/11/2205/s1, Details of calculations of Green’s function elements and densities of states.
