# Supplementary material for: Graphene-Based One-Dimensional Terahertz Phononic Crystal: Band Structures and Surface Modes
Source: Nanomaterials (Basel). 2020 Nov 5;10(11):2205. doi: 10.3390/nano10112205 (PMC7694383; doi:10.3390/nano10112205)
Supplement: Supplementary file 1 [file nanomaterials-10-02205-s001.pdf]

Article

Supplementary Material: Derivation of the total and local density of states using the Green's function method

Ilyasse Quotane [1], El Houssaine El Boudouti [1*] and Bahram Djafari-Rouhani [2]

1 Laboratoire de Physique de la Matière et de Rayonnement (LPMR), Département de Physique, Faculté des Sciences, Université Mohammed I, Oujda, Morocco ; q_ilyasse@yahoo.com (I.Q.), elboudouti@yahoo.fr (EL H.EL B.)

2 Institut d'Electronique, de Microélectronique et de Nanotechnologie (IEMN), UMR CNRS 8520, Département de Physique, Université de Lille, 59655 Villeneuve d'Ascq, France ; bahram.djafari-rouhani@univ-lille.fr (B.D-R)

* Correspondence: elboudouti@yahoo.fr (El H.El B.)

---

1. Expressions of the elements α_0, δ and γ in Eq. (6).

First of all, let us define the parameters a_i, b_i, d_i, e_i, f_i and q_i $(i = 1, 2)$ given in Eq. (2). If we omit the subscript i, these quantities are given by

$$a = FC_{44}(\alpha_1 - \zeta\alpha_3)(S_3C_1 - \zeta S_1C_3), \quad (1a)$$

$$b = FC_{33}(\alpha_3 - \zeta\alpha_1)(S_1C_3 - \zeta S_3C_1), \quad (1b)$$

$$q = Fk_{//}C_{44}\Big\{\zeta(2 + \tfrac{\alpha_1}{k_{//}\zeta_1} + \tfrac{\alpha_3}{k_{//}\zeta_3})(C_1C_3 - 1) - [1 + \tfrac{\alpha_3}{k_{//}\zeta_3} + \zeta^2(1 + \tfrac{\alpha_1}{k_{//}\zeta_1})]S_1S_3\Big\}, \quad (1c)$$

$$d = -FC_{44}(\alpha_1 - \zeta\alpha_3)(S_3 - \zeta S_1), \quad (1d)$$

$$e = -FC_{33}(\alpha_3 - \zeta\alpha_1)(S_1 - \zeta S_3), \quad (1e)$$

$$f = -F\frac{C_{44}}{\zeta_3}(\alpha_1 - \zeta\alpha_3)(C_1 - C_3), \quad (1f)$$

with

$$\zeta = \frac{\zeta_1}{\zeta_3} \quad (1g)$$

and

$$\zeta_i = \frac{C_{11}k_{//}^2 - C_{44}\alpha_i^2 - \rho\omega^2}{(C_{13} + C_{44})k_{//}\alpha_i} = \frac{(C_{13} + C_{44})k_{//}\alpha_i}{\rho\omega^2 - C_{44}k_{//}^2 + C_{33}\alpha_i^2} \qquad i = 1, 3 \quad (1h)$$

$$\alpha_1^2 = 1/2[x + (x^2 - 4y^2)^{1/2}], \quad (1i)$$

$$\alpha_3^2 = 1/2[x - (x^2 - 4y^2)^{1/2}], \quad (1j)$$

where

$$x = -\delta_1^2 - \delta_3^2 - \frac{(C_{13}+C_{44})^2}{C_{33}C_{44}}k_{//}^2, \tag{1k}$$

$$y^2 = \delta_1^2\delta_3^2, \tag{1l}$$

$$\delta_1^2 = \frac{(\rho\omega^2 - C_{11}k_{//}^2)}{C_{44}}, \tag{1m}$$

$$\delta_3^2 = \frac{(\rho\omega^2 - C_{44}k_{//}^2)}{C_{33}}, \tag{1n}$$

$$F = -[S_1S_3(1+\zeta^2) - 2\zeta(C_1C_3 - 1)]^{-1}, \tag{1o}$$

$$S_i = sinh(\alpha_i d) \qquad i = 1,3 \tag{1p}$$

and

$$S_i = cosh(\alpha_i d) \qquad i = 1,3 \tag{1q}$$

The expressions of the elements α_0, δ and γ in Eq. (6), are given by

$$\alpha_0 = 4[d_1e_1d_2e_2 + (f_1f_1)^2 + f_2^2d_1e_1 + f_1^2d_2e_2], \tag{2}$$

$$\delta = -\frac{B}{\alpha_0}, \tag{3}$$

$$\gamma = \frac{C}{\alpha_0} - \frac{1}{2}, \tag{4}$$

with

$$\begin{aligned} B = {} & (a_1+a_2)[(b_1+b_2)f_1f_2 + (q_1-q_2)(f_1e_2 - f_2e_1) - (a_1+a_2)e_1e_2] - \\ & (q_1-q_2)\{[2(b_1+b_2)f_1d_2 - (q_1-q_2)(f_1f_2+e_1d_2) + (a_1+a_2)f_2e_1] \\ & - [2(b_1+b_2)f_2d_1 + (q_1-q_2)(f_1f_2+e_2d_1) + (a_1+a_2)f_1e_2]\} + \\ & d_1[-(b_1+b_2)^2d_2 + e_1e_2d_1 + d_2(e_1^2+e_2^2) - 2f_1f_2e_1 + e_2f_2^2 + e_2f_1^2] + \\ & d_2[e_1(e_2d_2+f_2^2) + f_1(e_1f_1 - 2f_2e_2)] - f_1[-(a_1+a_2)(b_1+b_2)f_2 + 2f_2(f_1f_2+f_2^2)], \end{aligned} \tag{5}$$

$$\begin{aligned} C = {} & (a_1+a_2)\{(b_1+b_2)[(a_1+a_2)(b_1+b_2) - (q_1-q_2)^2 + (f_1^2+f_2^2)] - (a_1+a_2)(e_1^2+e_2^2) + \\ & 2(q_1-q_2)(f_1e_1 - f_2e_2)\} - (q_1-q_2)\{(q_1-q_2)[(a_1+a_2)(b_1+b_2) - (q_1-q_2)^2] + \\ & (q_1-q_2)(f_1^2+f_2^2) + (b_1+b_2)(d_1f_1 - d_2f_2) - (q_1-q_2)(d_1e_1+d_2e_2) - \\ & (a_1+a_2)(f_1e_1 - f_2e_2)\} + d_1[-2(q_1-q_2)(b_1+b_2)f_1 + (q_1-q_2)^2e_1 - (b_1+b_2)^2d_1 + \\ & d_1(e_1^2+e_2^2) + d_2e_1e_2 + f_1(e_1f_1 - 2f_2e_2)] + d_2[(q_1-q_2)(b_1+b_2)f_2 + (q_1-q_2)^2e_2 - \\ & (b_1+b_2)^2d_2 + (b_1+b_2)(q_1-q_2)f_2 + e_2(e_1d_1 + f_2^2 + d_2(e_1^2+e_2^2) - 2f_1f_2e_1] - \\ & f_1[(q_1-q_2)^2f_1 - (a_1+a_2)(q_1-q_2)e_1 + (b_1+b_2)(q_1-q_2)d_1 + (a_1+a_2)(b_1+b_2)f_1 \\ & - e_1(f_1d_1 - f_2d_2) + f_2(d_1e_2 - 2f_1f_2) - f_1^3] + f_2[-(q_1-q_2)^2f_2 - (a_1+a_2)(q_1-q_2)e_2 \\ & + (b_1+b_2)(q_1-q_2)d_2 - (a_1+a_2)(b_1+b_2)f_2 - f_1(d_2e_1+d_1e_2) + f_2(2f_1^2 + d_2e_2 + f_2^2)], \end{aligned} \tag{6}$$

2. Expression of the 16 elements of the Green's function in the interfaces space of the infinite superlattice.

By adopting the following notations: $\eta = \eta_1 - \eta_2$ and $\frac{t^{|n-n'+\mu|+1}}{t^2-1} = \frac{t_1^{|n-n'+\mu|+1}}{t_1^2-1} - \frac{t_2^{|n-n'+\mu|+1}}{t_2^2-1}(\mu = -2,-1,0,1,2)$, we get

i)

$$g_{11}(n,1,-\frac{d_1}{2};n',1,-\frac{d_1}{2}) = \frac{2}{\alpha_0\eta}\{A_0\frac{t^{|n-n'|+1}}{t^2-1} + A_1\frac{t^{|n-n'+1|+1}}{t^2-1} + A_1\frac{t^{|n-n'-1|+1}}{t^2-1}\}, \tag{7a}$$

$$g_{31}(n,1,-\frac{d_1}{2};n',1,-\frac{d_1}{2}) = -\frac{2i}{\alpha_0\eta}\{Q_0\frac{t^{|n-n'|+1}}{t^2-1} + Q_1\frac{t^{|n-n'+1|+1}}{t^2-1} + Q_1'\frac{t^{|n-n'-1|+1}}{t^2-1}\}, \tag{7b}$$

$$g_{13}(n,1,-\frac{d_1}{2};n',1,-\frac{d_1}{2}) = \frac{2i}{\alpha_0\eta}\{Q_0\frac{t^{|n-n'|+1}}{t^2-1} + Q_1\frac{t^{|n-n'-1|+1}}{t^2-1} + Q_1'\frac{t^{|n-n'+1|+1}}{t^2-1}\}, \tag{7c}$$

$$g_{33}(n,1,-\frac{d_1}{2};n',1,-\frac{d_1}{2}) = \frac{2}{\alpha_0\eta}\{B_0\frac{t^{|n-n'|+1}}{t^2-1} + B_1\frac{t^{|n-n'+1|+1}}{t^2-1} + B_1\frac{t^{|n-n'-1|+1}}{t^2-1}\}, \tag{7d}$$

ii)

$$g_{11}(n,1,\frac{d_1}{2};n',1,-\frac{d_1}{2}) = \frac{2}{\alpha_0\eta}\{D_0\frac{t^{|n-n'|+1}}{t^2-1} + D_1\frac{t^{|n-n'+1|+1}}{t^2-1} + D_1'\frac{t^{|n-n'-1|+1}}{t^2-1} + D_2\frac{t^{|n-n'+2|+1}}{t^2-1}\}, \tag{8a}$$

$$g_{31}(n,1,-\frac{d_1}{2};n',1,-\frac{d_1}{2}) = -\frac{2i}{\alpha_0\eta}\{F_0\frac{t^{|n-n'|+1}}{t^2-1} + F_1\frac{t^{|n-n'+1|+1}}{t^2-1} + F_1'\frac{t^{|n-n'-1|+1}}{t^2-1} + F_2\frac{t^{|n-n'+2|+1}}{t^2-1}\}, \tag{8b}$$

$$g_{13}(n,1,-\frac{d_1}{2};n',1,-\frac{d_1}{2}) = -\frac{2i}{\alpha_0\eta}\{F_0\frac{t^{|n-n'|+1}}{t^2-1} + F_1\frac{t^{|n-n'+1|+1}}{t^2-1} + F_1'\frac{t^{|n-n'-1|+1}}{t^2-1} + F_2\frac{t^{|n-n'+2|+1}}{t^2-1}\}, \tag{8c}$$

$$g_{33}(n,1,-\frac{d_1}{2};n',1,-\frac{d_1}{2}) = \frac{2}{\alpha_0\eta}\{E_0\frac{t^{|n-n'|+1}}{t^2-1} + E_1\frac{t^{|n-n'+1|+1}}{t^2-1} + E_1'\frac{t^{|n-n'-1|+1}}{t^2-1} + E_2\frac{t^{|n-n'+2|+1}}{t^2-1}\}, \tag{8d}$$

iii)

$$g_{11}(n,1,-\frac{d_1}{2};n',1,\frac{d_1}{2}) = \frac{2}{\alpha_0\eta}\{D_0\frac{t^{|n-n'|+1}}{t^2-1} + D_1\frac{t^{|n-n'-1|+1}}{t^2-1} + D_1'\frac{t^{|n-n'+1|+1}}{t^2-1} + D_2\frac{t^{|n-n'-2|+1}}{t^2-1}\}, \tag{9a}$$

$$g_{31}(n,1,-\frac{d_1}{2};n',1,\frac{d_1}{2}) = \frac{2i}{\alpha_0\eta}\{F_0\frac{t^{|n-n'|+1}}{t^2-1} + F_1\frac{t^{|n-n'+1|+1}}{t^2-1} + F_1'\frac{t^{|n-n'+1|+1}}{t^2-1} + F_2\frac{t^{|n-n'-2|+1}}{t^2-1}\}, \tag{9b}$$

$$g_{13}(n,1,-\frac{d_1}{2};n',1,\frac{d_1}{2}) = \frac{2i}{\alpha_0\eta}\{F_0\frac{t^{|n-n'|+1}}{t^2-1} + F_1\frac{t^{|n-n'-1|+1}}{t^2-1} + F_1'\frac{t^{|n-n'+1|+1}}{t^2-1} + F_2\frac{t^{|n-n'-2|+1}}{t^2-1}\}, \tag{9c}$$

$$g_{33}(n,1,-\frac{d_1}{2};n',1,\frac{d_1}{2}) = \frac{2}{\alpha_0\eta}\{E_0\frac{t^{|n-n'|+1}}{t^2-1} + E_1\frac{t^{|n-n'-1|+1}}{t^2-1} + E_1'\frac{t^{|n-n'+1|+1}}{t^2-1} + E_2\frac{t^{|n-n'-2|+1}}{t^2-1}\}, \tag{9d}$$

iv)

$$g_{11}(n,1,\frac{d_1}{2};n',1,\frac{d_1}{2}) = \frac{2}{\alpha_0\eta}\{A_0\frac{t^{|n-n'|+1}}{t^2-1} + A_1\frac{t^{|n-n'+1|+1}}{t^2-1} + A_1\frac{t^{|n-n'-1|+1}}{t^2-1}\}, \tag{10a}$$

$$g_{31}(n,1,\frac{d_1}{2};n',1,\frac{d_1}{2}) = \frac{2i}{\alpha_0\eta}\{Q_0\frac{t^{|n-n'|+1}}{t^2-1} + Q_1\frac{t^{|n-n'-1|+1}}{t^2-1} + Q_1'\frac{t^{|n-n'+1|+1}}{t^2-1}\}, \tag{10b}$$

$$g_{13}(n,1,\frac{d_1}{2};n',1,\frac{d_1}{2}) = -\frac{2i}{\alpha_0\eta}\{Q_0\frac{t^{|n-n'|+1}}{t^2-1} + Q_1\frac{t^{|n-n'+1|+1}}{t^2-1} + Q_1'\frac{t^{|n-n'-1|+1}}{t^2-1}\}, \tag{10c}$$

$$g_{33}(n,1,\frac{d_1}{2};n',1,\frac{d_1}{2}) = \frac{2}{\alpha_0\eta}\{B_0\frac{t^{|n-n'|+1}}{t^2-1} + B_1\frac{t^{|n-n'+1|+1}}{t^2-1} + B_1\frac{t^{|n-n'-1|+1}}{t^2-1}\}, \tag{10d}$$

with

$$\begin{aligned} A_0 = (b_1+b_2)[(a_1+a_2)(b_1+b_2) - (q_1-q_2)^2 - f_1^2 - f_2^2] + \\ 2(q_1-q_2)(e_1f_1 - e_2f_2) - (a_1+a_2)(e_1^2+e_2^2), \end{aligned} \tag{11a}$$

$$A_1 = (q_1-q_2)(f_1e_2 - f_2e_1) + (b_1+b_2)f_1f_2 - (a_1+a_2)e_1e_2, \tag{11b}$$

$$\begin{aligned} Q_0 = -(q_1-q_2)[(a_1+a_2)(b_1+b_2) - (q_1-q_2)^2] - (b_1+b_2)(d_1f_1 - d_2f_2) + \\ (q_1-q_2)(d_1e_1+d_2e_2) - (q_1-q_2)(f_1^2+f_2^2) + (a_1+a_2)(f_1e_1 - f_2e_2), \end{aligned} \tag{11c}$$

$$Q_1 = -[(b_1+b_2)d_2f_1 - (q_1-q_2)(d_2e_1 + f_1f_2) + (a_1+a_2)f_2e_1], \tag{11d}$$

$$Q_1' = (b_1+b_2)d_1f_2 + (q_1-q_2)(d_1e_2 + f_1f_2) + (a_1+a_2)f_1e_2, \tag{11e}$$

$$\begin{aligned} B_0 = (a_1+a_2)[(a_1+a_2)(b_1+b_2) - (q_1-q_2)^2 - (f_1^2+f_2^2)] - (b_1+b_2)(d_1^2+d_2^2) \\ -2(q_1-q_2)(f_1d_1 - f_2d_2), \end{aligned} \tag{11f}$$

$$B_1 = -[(b_1+b_2)d_1d_2 + (q_1-q_2)(f_1d_2 - f_2d_1) - (a_1+a_2)f_1f_2], \tag{11g}$$

$$\begin{aligned} D_0 = -(q_1-q_2)[2(b_1+b_2)f_1 - (q_1-q_2)e_1] - (b_1+b_2)^2d_1 + \\ d_1(e_1^2+e_2^2) + e_1(d_2e_2 + f_1^2) - 2f_1f_2e_2, \end{aligned} \tag{11h}$$

$$\begin{aligned} D_1 = (q_1-q_2)[2(b_1+b_2)f_2 + (q_1-q_2)e_2] - (b_1+b_2)e_2] - (b_1+b_2)^2d_2 + (e_1^2+e_2^2)d_2 + \\ e_1(d_1e_2 - 2f_1f_2) + e_2f_2^2, \end{aligned} \tag{11i}$$

$$D_1' = e_2(d_1e_1 + f_1^2), \tag{11j}$$

$$D_2 = e_1(d_2e_2 + f_2^2), \tag{11k}$$

$$\begin{aligned} F_0 = -(q_1-q_2)[(q_1-q_2)f_1 - (a_1+a_2)e_1 + (b_1+b_2)d_1] + d_1(f_1e_1 - f_2e_2) - \\ d_2f_2e_1 + f_1[-(b_1+b_2)(a_1+a_2) + f_1^2 + 2f_2^2], \end{aligned} \tag{11l}$$

$$\begin{aligned} F_1 = (q_1-q_2)[(q_1-q_2)f_2 + (a_1+a_2)e_2 - (b_1+b_2)d_2] + d_2(f_1e_1 - f_2e_2) - \\ d_1f_1e_2 + f_2[(b_1+b_2)(a_1+a_2) - f_2^2 - 2f_1^2], \end{aligned} \tag{11m}$$

$$F_1' = -f_2(e_1d_1 + f_1^2), \tag{11n}$$

$$F_2 = -f_1(e_2d_2 + f_2^2), \tag{11o}$$

$$\begin{aligned} E_0 = (q_1-q_2)[(a_1+a_2)f_1 + (q_1-q_2)d_1 + (a_1+a_2)f_1] - (a_1+a_2)^2e_1 + \\ (d_1^2+d_2^2)e_1 + d_1(e_1d_1 + f_1^2) - 2f_1f_2d_2, \end{aligned} \tag{11p}$$

$$\begin{aligned} E_1 = (q_1-q_2)[-(a_1+a_2)f_2 + (q_1-q_2)d_2 + (a_1+a_2)f_2] - (a_1+a_2)^2e_2 + \\ (d_1^2+d_2^2)e_2 + d_2(e_2d_2 + f_2^2) - 2f_1f_2d_1, \end{aligned} \tag{11q}$$

$$E_1' = d_2(e_1d_1 + f_1^2), \tag{11r}$$

$$E_2 = d_1(e_2d_2 + f_2^2), \tag{11s}$$

3. Expression of matrices: $G_i(x_3, x_3'), G_i^{-1}(M_m, M_m), G_i(x_3, M_m)$ **and** $G_i(M_m, x_3')$ **in Eqs. (14) and (19).**

For simplicity, we will omit the index "i" in the following expressions. Let us first recall the expression of the bulk Green's function

$$G(x_3,x_3') = \begin{pmatrix} \frac{1}{F\zeta_1}[-e^{-\alpha_1|x_3-x_3'|}+\zeta e^{-\alpha_3|x_3-x_3'|}] & \frac{i}{F}sgn(x_3-x_3')[-e^{-\alpha_1|x_3-x_3'|}+e^{-\alpha_3|x_3-x_3'|}] \\ \frac{i}{F}sgn(x_3-x_3')[-e^{-\alpha_1|x_3-x_3'|}+e^{-\alpha_3|x_3-x_3'|}] & \frac{\zeta_3}{F}[\zeta e^{-\alpha_1|x_3-x_3'|}-e^{-\alpha_3|x_3-x_3'|} \end{pmatrix}. \tag{12}$$

where

$$F = \Big[\frac{k_{//}(C_{44}+C_{13})}{2C_{44}C_{33}(\alpha_1^2-\alpha_2^2)}\Big]^{-1}, \tag{13}$$

α_1 , α_3, ζ_1, ζ_3 and ζ are defined From Eq. (1) above.

$G^{-1}(M_m,M_m)$ is obtained from Eq. (12) and can be written as

$$G^{-1}(M_m,M_m) = \begin{pmatrix} r & is & u & -iv \\ ip & t & iz & w \\ u & iv & r & -is \\ -iz & w & -ip & t \end{pmatrix}. \tag{14}$$

where

$$r = \frac{\zeta_3}{2D_s}(\beta_3^- - \zeta\beta_1^-) + \frac{\zeta_3}{2D_{As}}(\beta_3^+ - \zeta\beta_1^+), \tag{15a}$$

$$s = \frac{1}{2D_s}(\beta_3^+ - \beta_1^+) + \frac{1}{2D_{As}}(\beta_3^- - \beta_1^-), \tag{15b}$$

$$p = \frac{1}{2D_s}(\beta_3^- - \beta_1^-) + \frac{1}{2D_{As}}(\beta_3^+ - \beta_1^+), \tag{15c}$$

$$t = \frac{1}{2D_s\zeta_1}(\beta_1^+ - \zeta\beta_3^+) + \frac{1}{2D_{As}\zeta_1}(\beta_1^- - \zeta\beta_3^-), \tag{15d}$$

$$u = \frac{\zeta_3}{4D_s}(\beta_3^- - \zeta\beta_1^-) - \frac{\zeta_3}{4D_{As}}(\beta_3^+ - \zeta\beta_1^+), \tag{15e}$$

$$v = \frac{1}{2D_s}(\beta_3^+ - \beta_1^+) - \frac{1}{2D_{As}}(\beta_3^- - \beta_1^-), \tag{15f}$$

$$z = \frac{1}{2D_s}(\beta_3^- - \beta_1^-) - \frac{1}{2D_{As}}(\beta_3^+ - \beta_1^+), \tag{15g}$$

$$w = \frac{-1}{2D_s\zeta_1}(\beta_1^+ - \zeta\beta_3^+) + \frac{1}{2D_{As}\zeta_1}(\beta_1^- - \zeta\beta_3^-), \tag{15h}$$

with

$$D_s = -\frac{1}{F}(\frac{1-\zeta}{\zeta})[\beta_1^+\beta_3^- - \zeta\beta_3^+\beta_1^-], \tag{16a}$$

$$D_{As} = -\frac{1}{F}(\frac{1-\zeta}{\zeta})[\beta_1^-\beta_3^+ - \zeta\beta_3^-\beta_1^+], \tag{16b}$$

$$\beta_1^+ = 1+e^{-\alpha_1 d_1},\ \beta_1^- = 1-e^{-\alpha_1 d_1},\ \beta_3^+ = 1+e^{-\alpha_3 d_1},\ \beta_3^- = 1-e^{-\alpha_3 d_1}, \tag{16c}$$

$G(x_3,M_m)$ and $G(M_m,x_3')$ can be calculated from Eq. (12) as follows

$$G(x_3,M_m) = (e^{-\alpha_1(\frac{d_1}{2}+x_3)}Q_1^- + e^{-\alpha_3(\frac{d_1}{2}+x_3)}Q_3^-\ ,\ e^{-\alpha_1(\frac{d_1}{2}-x_3)}Q_1^+ + e^{-\alpha_3(\frac{d_1}{2}-x_3)}Q_3^+), \tag{17a}$$

$$G(M_{m'},x_3') = \begin{pmatrix} e^{-\alpha_1(\frac{d_1}{2}+x_3')}Q_1^+ + e^{-\alpha_3(\frac{d_1}{2}+x_3')}Q_3^+ \\ e^{-\alpha_1(\frac{d_1}{2}-x_3')}Q_1^- + e^{-\alpha_3(\frac{d_1}{2}-x_3')}Q_3^- \end{pmatrix}. \tag{17b}$$

$G(x_3, M_m)$ and $G(M_m, x_3')$ are (2×4) and (4×2) matrices respectively, with

$$Q_1^+ = \frac{1}{F}\begin{pmatrix} -\frac{1}{\zeta_1} & i \\ i & \zeta_1 \end{pmatrix},\quad Q_3^+ = \frac{1}{F}\begin{pmatrix} \frac{1}{\zeta_3} & -i \\ -i & -\zeta_3 \end{pmatrix}, \tag{18a}$$

$$Q_1^- = \frac{1}{F}\begin{pmatrix} -\frac{1}{\zeta_1} & -i \\ -i & \zeta_1 \end{pmatrix},\quad Q_3^- = \frac{1}{F}\begin{pmatrix} \frac{1}{\zeta_3} & i \\ i & -\zeta_3 \end{pmatrix}, \tag{18b}$$

where F is given by Eq. (13)

4. Expressions of the elements of the surface response operator A in Eq. (3) of the article.

According to Eq. (16) in the article, we have

$$A(0,1,\frac{d_1}{2};n',1\pm\frac{d_1}{2}) = V(0,1,\frac{d_1}{2};0,1,\frac{d_1}{2})g(0,1,\frac{d_1}{2};n',1\pm\frac{d_1}{2})+ V(0,1,\frac{d_1}{2};1,1,-\frac{d_1}{2})g(1,1,-\frac{d_1}{2};n',1\pm\frac{d_1}{2}), \tag{19}$$

where the terms $V(0,1,\frac{d_1}{2};0,1,\frac{d_1}{2})$ and $V(0,1,\frac{d_1}{2};1,1,-\frac{d_1}{2})$ are obtained from Eqs. (15) and (2) in the article, respectively, namely

$$V(0,1,\frac{d_1}{2};0,1,\frac{d_1}{2}) = -A_2 = -\begin{pmatrix} a_2 & iq_2 \\ -iq_2 & b_2 \end{pmatrix}, \text{and}$$

$$V(0,1,\frac{d_1}{2};1,1,-\frac{d_1}{2}) = -B_2 = -\begin{pmatrix} d_2 & if_2 \\ if_2 & e_2 \end{pmatrix}, \tag{20}$$

while the terms $g(0,1,\frac{d_1}{2};n',1,\pm\frac{d_1}{2})$ and $g(1,1,-\frac{d_1}{2};n',1,\pm\frac{d_1}{2})$ are obtained from Eqs. (7)-(10), namely

$$g(0,1,\frac{d_1}{2};n',1,-\frac{d_1}{2}) = \frac{2}{\alpha_0\eta}\times \begin{bmatrix} D_0\frac{t^{-n'+1}}{t^2-1}+D_1\frac{t^{-n'+2}}{t^2-1}+D_1'\frac{t^{|1+n'|+1}}{t^2-1}+D_2\frac{t^{-n'+3}}{t^2-1} & -i\{F_0\frac{t^{-n'+1}}{t^2-1}+F_1\frac{t^{-n'+2}}{t^2-1}+F_1'\frac{t^{|1+n'|+1}}{t^2-1}+F_2\frac{t^{-n'+3}}{t^2-1}\} \\ -i\{F_0\frac{t^{-n'+1}}{t^2-1}+F_1\frac{t^{-n'+2}}{t^2-1}+F_1'\frac{t^{|1+n'|+1}}{t^2-1}+F_2\frac{t^{-n'+3}}{t^2-1}\} & E_0\frac{t^{-n'+1}}{t^2-1}+E_1\frac{t^{-n'+2}}{t^2-1}+E_1'\frac{t^{|1+n'|+1}}{t^2-1}+E_2\frac{t^{-n'+3}}{t^2-1} \end{bmatrix}, \tag{21}$$

$$g(0,1,\frac{d_1}{2};n',1,\frac{d_1}{2}) = \frac{2}{\alpha_0\eta}\times \begin{bmatrix} A_0\frac{t^{-n'+1}}{t^2-1}+A_1\frac{t^{-n'+2}}{t^2-1}+A_1\frac{t^{|1+n'|+1}}{t^2-1} & -i\{Q_0\frac{t^{-n'+1}}{t^2-1}+Q_1\frac{t^{-n'+2}}{t^2-1}+Q_1'\frac{t^{|1+n'|+1}}{t^2-1}\} \\ i\{Q_0\frac{t^{-n'+1}}{t^2-1}+Q_1\frac{t^{|1+n'|+1}}{t^2-1}+Q_1'\frac{t^{-n'+2}}{t^2-1}\} & B_0\frac{t^{-n'+1}}{t^2-1}+B_1\frac{t^{-n'+2}}{t^2-1}+B_1\frac{t^{|1+n'|+1}}{t^2-1} \end{bmatrix}, \tag{22}$$

$$g(1,1,-\frac{d_1}{2};n',1,-\frac{d_1}{2}) = \frac{2}{\alpha_0\eta}\times \begin{bmatrix} A_0\frac{t^{-n'+2}}{t^2-1}+A_1\frac{t^{-n'+3}}{t^2-1}+A_1\frac{t^{-n'+1}}{t^2-1} & i\{Q_0\frac{t^{-n'+2}}{t^2-1}+Q_1\frac{t^{-n'+1}}{t^2-1}+Q_1'\frac{t^{-n'+3}}{t^2-1}\} \\ -i\{Q_0\frac{t^{-n'+2}}{t^2-1}+Q_1\frac{t^{-n'+3}}{t^2-1}+Q_1'\frac{t^{-n'+1}}{t^2-1}\} & B_0\frac{t^{-n'+2}}{t^2-1}+B_1\frac{t^{-n'+3}}{t^2-1}+B_1\frac{t^{-n'+1}}{t^2-1} \end{bmatrix}, \tag{23}$$

$$g(1,1,-\frac{d_1}{2};n',1,\frac{d_1}{2}) = \frac{2}{\alpha_0\eta}\times$$
$$\begin{bmatrix} D_0\frac{t^{-n'+2}}{t^2-1} + D_1\frac{t^{-n'+1}}{t^2-1} + D_1'\frac{t^{-n'+3}}{t^2-1} + D_2\frac{t^{|1+n'|+1}}{t^2-1} & i\{F_0\frac{t^{-n'+2}}{t^2-1} + F_1\frac{t^{-n'+1}}{t^2-1} + F_1'\frac{t^{-n'+3}}{t^2-1} + F_2\frac{t^{|1+n'|+1}}{t^2-1}\} \\ i\{F_0\frac{t^{-n'+2}}{t^2-1} + F_1\frac{t^{-n'+1}}{t^2-1} + F_1'\frac{t^{-n'+3}}{t^2-1} + F_2\frac{t^{|1+n'|+1}}{t^2-1}\} & E_0\frac{t^{-n'+2}}{t^2-1} + E_1\frac{t^{-n'+1}}{t^2-1} + E_1'\frac{t^{-n'+3}}{t^2-1} + E_2\frac{t^{|1+n'|+1}}{t^2-1} \end{bmatrix}, \quad (24)$$

5. Expressions of the elements of the Green's function in the interfaces space of the semi-infinite superlattice

In order to facilitate the calculation of the Green's function elements in the interface space, we should distinguish two cases: $n,n' \leq -1$ and $n = n' = 0$.

i) Case $n,n' \leq -1$.

The Green's function elements in the interface space are given by

$$d(n,1,\pm\frac{d_1}{2};n',1,\pm\frac{d_1}{2}) = g(n,1,\pm\frac{d_1}{2};n',1,\pm\frac{d_1}{2}) - g(n,1,\pm\frac{d_1}{2};0,1,\frac{d_1}{2})\Delta^{-1}(0,1,\frac{d_1}{2};0,1,\frac{d_1}{2})A(0,1,\frac{d_1}{2};n',1,\pm\frac{d_1}{2}), \quad (25)$$

where $\Delta^{-1}(0,1,\frac{d_1}{2};0,1,\frac{d_1}{2})$ is the inverse of the matrix $\Delta(0,1,\frac{d_1}{2};0,1,\frac{d_1}{2})$:

$$\Delta(0,1,\frac{d_1}{2};0,1,\frac{d_1}{2}) = I + A(0,1,\frac{d_1}{2};0,1,\frac{d_1}{2}), \quad (26)$$

The elements of the operator $A(0,1,\frac{d_1}{2};0,1,\frac{d_1}{2})$ are obtained from Eqs. (19)-(24). $g(n,1,\pm\frac{d_1}{2};n',1,\pm\frac{d_1}{2})$ is given by Eqs. (7)-(10), whereas $g(n,1,\pm\frac{d_1}{2};0,1,\frac{d_1}{2})$ can be obtained from Eqs. (9)-(10), namely

$$g(n,1,-\frac{d_1}{2};0,1,\frac{d_1}{2}) = \frac{2}{\alpha_0(\eta_1-\eta_2)}\{\Psi_0\frac{t^{-n}}{t^2-1} + \Psi_1\frac{t^{1-n}}{t^2-1} + \Psi_2\frac{t^{2-n}}{t^2-1} + \Psi_3\frac{t^{3-n}}{t^2-1}\}, \quad (27)$$

$$g(n,1,\frac{d_1}{2};0,1,\frac{d_1}{2}) = \frac{2}{\alpha_0(\eta_1-\eta_2)}\{\Psi_0'\frac{t^{-n}}{t^2-1} + \Psi_1'\frac{t^{1-n}}{t^2-1} + \Psi_2'\frac{t^{2-n}}{t^2-1}\}, \quad (28)$$

with

$$\Psi_0 = \begin{pmatrix} D_1' & iF_1' \\ iF_1' & E_1' \end{pmatrix}, \Psi_1 = \begin{pmatrix} D_0 & iF_0 \\ iF_0 & E_0 \end{pmatrix}, \Psi_2 = \begin{pmatrix} D_1 & iF_1 \\ iF_1 & E_1 \end{pmatrix}, \Psi_3 = \begin{pmatrix} D_2 & iF_2 \\ iF_2 & E_2 \end{pmatrix}, \quad (29)$$

$$\Psi_0' = \begin{pmatrix} A_1 & -iQ_1 \\ iQ_1' & B_1 \end{pmatrix}, \Psi_1' = \begin{pmatrix} A_0 & -iQ_0 \\ iQ_0 & B_0 \end{pmatrix}, \Psi_2' = \begin{pmatrix} A_1 & -iQ_1' \\ iQ_1 & B_1 \end{pmatrix}, \quad (30)$$

$A(0,1,\frac{d_1}{2};n',1,\pm\frac{d_1}{2})$ is obtained from Eqs. (19)-(24), namely

$$A(0,1,\frac{d_1}{2};n',1,-\frac{d_1}{2}) = -\frac{2}{\alpha_0(\eta_1-\eta_2)}\{X_0\frac{t^{-n'}}{t^2-1} + X_1\frac{t^{1-n'}}{t^2-1} + X_2\frac{t^{2-n'}}{t^2-1} + X_3\frac{t^{3-n'}}{t^2-1}\}, \quad (31)$$

$$A(0,1,\frac{d_1}{2};n',1,\frac{d_1}{2}) = -\frac{2}{\alpha_0(\eta_1-\eta_2)}\{X_0'\frac{t^{-n'}}{t^2-1} + X_1'\frac{t^{1-n'}}{t^2-1} + X_2'\frac{t^{2-n'}}{t^2-1} + X_3'\frac{t^{3-n'}}{t^2-1}\}, \quad (32)$$

with

$$X_0 = \begin{pmatrix} a_2 D_1' + q_2 F_1' & i(-a_2 F_1' + q_2 E_1') \\ i(-q_2 D_1' - b_2 F_1') & -q_2 F_1' + b_2 E_1' \end{pmatrix}, \tag{33a}$$

$$X_1 = \begin{pmatrix} a_2 D_0 + q_2 F_0 + d_2 A_1 + f_2 Q_1' & i(-a_2 F_0 + q_2 E_0 + d_2 Q_1 + f_2 B_1) \\ i(-q_2 D_0 - b_2 F_0 + f_2 A_1 - e_2 Q_1') & -q_2 F_0 + b_2 E_0 - f_2 Q_1 + e_2 B_1 \end{pmatrix}, \tag{33b}$$

$$X_2 = \begin{pmatrix} a_2 D_1 + q_2 F_1 + d_2 A_0 + f_2 Q_0 & i(-a_2 F_1 + q_2 E_1 + d_2 Q_0 + f_2 B_0) \\ i(-q_2 D_1 - b_2 F_1 + f_2 A_0 - e_2 Q_0) & -q_2 F_1 + b_2 E_1 - f_2 Q_0 + e_2 B_0 \end{pmatrix}, \tag{33c}$$

$$X_3 = \begin{pmatrix} a_2 D_2 + q_2 F_2 + d_2 A_1 + f_2 Q_1 & i(-a_2 F_2 + q_2 E_2 + d_2 Q_1' + f_2 B_1) \\ i(-q_2 D_2 - b_2 F_2 + f_2 A_1 - e_2 Q_1) & -q_2 F_2 + b_2 E_2 - f_2 Q_1' + e_2 B_1 \end{pmatrix}, \tag{33d}$$

$$X_0' = \begin{pmatrix} a_2 A_1 - q_2 Q_1 + d_2 D_2 - f_2 F_2 & i(-a_2 Q_1' + q_2 B_1 + d_2 F_2 + f_2 E_2) \\ i(-q_2 A_1 + b_2 Q_1 + f_2 D_2 + e_2 F_2) & -q_2 Q_1' + b_2 B_1 - f_2 F_2 + e_2 E_2 \end{pmatrix}, \tag{34a}$$

$$X_1' = \begin{pmatrix} a_2 A_0 - q_2 Q_0 + d_2 D_1 - f_2 F_1 & i(-a_2 Q_0 + q_2 B_0 + d_2 F_1 + f_2 E_1) \\ i(-q_2 A_0 + b_2 Q_0 + f_2 D_1 + e_2 F_1) & -q_2 Q_0 + b_2 B_0 - f_2 F_1 + e_2 E_1 \end{pmatrix}, \tag{34b}$$

$$X_2' = \begin{pmatrix} a_2 A_1 - q_2 Q_1' + d_2 D_0 - f_2 F_0 & i(-a_2 Q_1 + q_2 B_1 + d_2 F_0 + f_2 E_0) \\ i(-q_2 A_1 + b_2 Q_1' + f_2 D_0 + e_2 F_0) & -q_2 Q_1 + b_2 B_1 - f_2 F_0 + e_2 E_0 \end{pmatrix}, \tag{34c}$$

$$X_3' = \begin{pmatrix} d_2 D_1' - f_2 F_1' & i(d_2 F_1' + f_2 E_1') \\ i(f_2 D_1' + e_2 F_1') & -f_2 F_1' + e_2 E_1' \end{pmatrix}, \tag{34d}$$

$A_0, A_1, B_0, B_1, Q_0, Q_1, Q_1', D_0, D_1', D_2, F_0, F_1, F_1', F_2, E_0, E_1, E_1'$ and E_2 are defined by Eq. (11).

Therefore, the elements of the Green's function in the interface space are given by

$$\begin{gathered} d(n,1,-\tfrac{d_1}{2};n',1,-\tfrac{d_1}{2}) = g(n,1,-\tfrac{d_1}{2};n',1,-\tfrac{d_1}{2}) + \frac{4}{\alpha_0^2(\eta_1-\eta_2)^2}\times \\ \{[\Psi_0 \tfrac{t^{-n}}{t^2-1} + \Psi_1 \tfrac{t^{1-n}}{t^2-1} + \Psi_2 \tfrac{t^{2-n}}{t^2-1} + \Psi_3 \tfrac{t^{3-n}}{t^2-1}]\Delta^{-1}[X_0 \tfrac{t^{-n}}{t^2-1} + X_1 \tfrac{t^{1-n}}{t^2-1} + X_2 \tfrac{t^{2-n}}{t^2-1} + X_3 \tfrac{t^{3-n}}{t^2-1}]\}, \end{gathered} \tag{35a}$$

$$\begin{gathered} d(n,1,-\tfrac{d_1}{2};n',1,\tfrac{d_1}{2}) = g(n,1,-\tfrac{d_1}{2};n',1,\tfrac{d_1}{2}) + \frac{4}{\alpha_0^2(\eta_1-\eta_2)^2}\times \\ \{[\Psi_0 \tfrac{t^{-n}}{t^2-1} + \Psi_1 \tfrac{t^{1-n}}{t^2-1} + \Psi_2 \tfrac{t^{2-n}}{t^2-1} + \Psi_3 \tfrac{t^{3-n}}{t^2-1}]\Delta^{-1}[X_0' \tfrac{t^{-n}}{t^2-1} + X_1' \tfrac{t^{1-n}}{t^2-1} + X_2' \tfrac{t^{2-n}}{t^2-1} + X_3' \tfrac{t^{3-n}}{t^2-1}]\}, \end{gathered} \tag{35b}$$

$$\begin{gathered} d(n,1,\tfrac{d_1}{2};n',1,-\tfrac{d_1}{2}) = g(n,1,\tfrac{d_1}{2};n',1,-\tfrac{d_1}{2}) + \frac{4}{\alpha_0^2(\eta_1-\eta_2)^2}\times \\ \{[\Psi_0' \tfrac{t^{-n}}{t^2-1} + \Psi_1' \tfrac{t^{1-n}}{t^2-1} + \Psi_2' \tfrac{t^{2-n}}{t^2-1}]\Delta^{-1}[X_0 \tfrac{t^{-n}}{t^2-1} + X_1 \tfrac{t^{1-n}}{t^2-1} + X_2 \tfrac{t^{2-n}}{t^2-1} + X_3 \tfrac{t^{3-n}}{t^2-1}]\}, \end{gathered} \tag{35c}$$

$$\begin{gathered} d(n,1,\tfrac{d_1}{2};n',1,\tfrac{d_1}{2}) = g(n,1,\tfrac{d_1}{2};n',1,\tfrac{d_1}{2}) + \frac{4}{\alpha_0^2(\eta_1-\eta_2)^2}\times \\ \{[\Psi_0' \tfrac{t^{-n}}{t^2-1} + \Psi_1' \tfrac{t^{1-n}}{t^2-1} + \Psi_2' \tfrac{t^{2-n}}{t^2-1}]\Delta^{-1}[X_0' \tfrac{t^{-n}}{t^2-1} + X_1' \tfrac{t^{1-n}}{t^2-1} + X_2' \tfrac{t^{2-n}}{t^2-1} + X_3' \tfrac{t^{3-n}}{t^2-1}]\}, \end{gathered} \tag{35d}$$

Recall that the term $\frac{t^{i-n}}{t^2-1}$ designates the quantity $\frac{t_1^{i-n}}{t_1^2-1} - \frac{t_2^{i-n}}{t_2^2-1}$, where t_1 and t_2 are defined by Eq. (8) of the article.

ii) Case $n = n' = 0$.

The Green's function elements in the interface space are given by

$$\begin{aligned} d(0,1,\pm\frac{d_1}{2};0,1,\pm\frac{d_1}{2}) &= g(0,1,\pm\frac{d_1}{2};0,1,\pm\frac{d_1}{2}) - \\ & g(0,1,\pm\frac{d_1}{2};0,1,\pm\frac{d_1}{2})\Delta^{-1}(0,1,\frac{d_1}{2};0,1,\frac{d_1}{2})A(0,1,\frac{d_1}{2};0,1,\pm\frac{d_1}{2}), \end{aligned} \tag{36}$$

where $g(0,1,\pm\frac{d_1}{2};0,1,\pm\frac{d_1}{2})$ is obtained from Eqs. (7)-(10) for $n = n' = 0$. $A(0,1,\frac{d_1}{2};0,1,\pm\frac{d_1}{2})$ is obtained from Eqs. (19)-(24) for $n' = 0$

6. Calculation of local and total densities of states

i) Local density of states

The local density of states on the plan (n, i, x_3) is given by

$$n_\alpha(\omega^2, k_{//}; n, ix_3) = -\frac{1}{\pi} Im\Big(d_{\alpha\alpha}(\omega^2, k_{//}|n, i, x_3; n, i, x_3)\Big) \quad (\alpha = 1, 3), \tag{37}$$

where $d_{\alpha\alpha}(\omega^2)$ designates the components 11 and 33 of the Green's function

$$d(n, i, x_3; n', i', x_3') = \delta_{nn'}\delta_{ii'}[G_i(x_3, x_3') - G_i(x_3, M_m)G_i^{-1}(M_m, M_{m'})G_{i'}(M_{m'}, x_3')] + \\ G_i(x_3, M_m)G_i^{-1}(M_m, M_m)d(M_m, M_{m'})G_{i'}^{-1}(M_{m'}, M_{m'})G_{i'}(M_{m'}, x_3'), \tag{38}$$

where the different matrices $G_i(x_3, x_3), G_{i'}^{-1}(M_m, M_m), G_i(x_3, M_m), G_i(M_m, x_3)$ and $d(M_m, M_m)$ are defined by Eqs. (12), (14), (17a), (17b) and (35.c) respectively.

ii) Total density of states

The calculation of the total density of states is obtained from the Eqs. (24.a) and (25.a) of the article. We first show how to obtain the variation of the total density of states of the media 1 of the semi-infinite superlattice

$$\Delta n_1(\omega^2) = -\frac{\rho_1}{\pi} Im\Big(tr \\ \int_{-\frac{d_1}{2}}^{\frac{d_1}{2}} G_1(x_3, M_m)G_1^{-1}(M_m, M_m) \sum_{n=-\infty}^{0} [d(M_m, M_m) - g(M_m, M_m)]G_1^{-1}(M_m, M_m)G_1(M_m, x_3)dx_3\Big), \tag{39}$$

In Eq. (39), we can first start by performing the summation $\sum_{n=-\infty}^{-1}[d(M_m, M_m) - g(M_m, M_m)]$.

From Eq. (35a), we get after summation over $n \leq -1$, the quantity

$$\sum_{-\infty}^{-1}[d(n,1,-\frac{d_1}{2},n,1,-\frac{d_1}{2}) - g(n,1,-\frac{d_1}{2},n,1,-\frac{d_1}{2})] = \frac{4}{\alpha_0^2(\eta_1-\eta_2)^2}\times$$
$$\{\Psi_0\Delta^{-1}X_0[-\frac{t_1^2}{(t_1^2-1)^3} + \frac{2t_1t_2}{(t_1^2-1)(t_2^2-1)(t_1t_2-1)} - \frac{t_2^2}{(t_2^2-1)^3}]+$$
$$\Psi_1\Delta^{-1}X_1[-\frac{t_1^4}{(t_1^2-1)^3} + \frac{2t_1^2t_2^2}{(t_1^2-1)(t_2^2-1)(t_1t_2-1)} - \frac{t_2^4}{(t_2^2-1)^3}]+$$
$$\Psi_2\Delta^{-1}X_2[-\frac{t_1^6}{(t_1^2-1)^3} + \frac{2t_1^3t_2^3}{(t_1^2-1)(t_2^2-1)(t_1t_2-1)} - \frac{t_2^6}{(t_2^2-1)^3}]+$$
$$\Psi_3\Delta^{-1}X_3[-\frac{t_1^8}{(t_1^2-1)^3} + \frac{2t_1^4t_2^4}{(t_1^2-1)(t_2^2-1)(t_1t_2-1)} - \frac{t_2^8}{(t_2^2-1)^3}]+$$
$$(\Psi_0\Delta^{-1}X_1+\Psi_1\Delta^{-1}X_0)[-\frac{t_1^3}{(t_1^2-1)^3} + \frac{t_1t_2(t_1+t_2)}{(t_1^2-1)(t_2^2-1)(t_1t_2-1)} - \frac{t_2^3}{(t_2^2-1)^3}]+$$
$$(\Psi_0\Delta^{-1}X_2+\Psi_2\Delta^{-1}X_0)[-\frac{t_1^4}{(t_1^2-1)^3} + \frac{t_1t_2(t_1^2+t_2^2)}{(t_1^2-1)(t_2^2-1)(t_1t_2-1)} - \frac{t_2^4}{(t_2^2-1)^3}]+$$
$$(\Psi_0\Delta^{-1}X_3+\Psi_3\Delta^{-1}X_0)[-\frac{t_1^5}{(t_1^2-1)^3} + \frac{t_1t_2(t_1^3+t_2^3)}{(t_1^2-1)(t_2^2-1)(t_1t_2-1)} - \frac{t_2^5}{(t_2^2-1)^3}]+$$
$$(\Psi_1\Delta^{-1}X_2+\Psi_2\Delta^{-1}X_1)[-\frac{t_1^5}{(t_1^2-1)^3} + \frac{t_1^2t_2^2(t_1+t_2)}{(t_1^2-1)(t_2^2-1)(t_1t_2-1)} - \frac{t_2^5}{(t_2^2-1)^3}]+$$
$$(\Psi_1\Delta^{-1}X_3+\Psi_3\Delta^{-1}X_1)[-\frac{t_1^6}{(t_1^2-1)^3} + \frac{t_1^2t_2^2(t_1^2+t_2^2)}{(t_1^2-1)(t_2^2-1)(t_1t_2-1)} - \frac{t_2^6}{(t_2^2-1)^3}]+$$
$$(\Psi_2\Delta^{-1}X_3+\Psi_3\Delta^{-1}X_2)[-\frac{t_1^7}{(t_1^2-1)^3} + \frac{t_1^3t_2^3(t_1+t_2)}{(t_1^2-1)(t_2^2-1)(t_1t_2-1)} - \frac{t_2^7}{(t_2^2-1)^3}]\}, \quad (40)$$

Similarly, from Eq. (35b), we get the quantity $\sum_{n=-\infty}^{-1}[d(n,1,-\frac{d_1}{2},n,1,\frac{d_1}{2}) - g(n,1,-\frac{d_1}{2},n,1,\frac{d_1}{2})]$ which can be written as the sum of 16 terms similarly to Eq. (40) by simply replacing X_0, X_1, X_2, X_3 by X'_0, X'_1, X'_2, X'_3 respectively.

Likewise, from Eq. (35.c), we get the quantity $\sum_{n=-\infty}^{-1}[d(n,1,\frac{d_1}{2},n,1,-\frac{d_1}{2}) - g(n,1,\frac{d_1}{2},n,1,-\frac{d_1}{2})]$ which can be written as the sum of 12 terms similarly to Eq. (40) by replacing just $\Psi_0, \Psi_1, \Psi_2, \Psi_3$ by $\Psi'_0, \Psi'_1, \Psi'_2, \Psi'_3$ respectively.

Also, from Eq. (35d), we get the quantity $\sum_{n=-\infty}^{-1}[d(n,1,\frac{d_1}{2},n,1,\frac{d_1}{2}) - g(n,1,\frac{d_1}{2},n,1,\frac{d_1}{2})]$ which can be written as the sum of 12 terms similarly to Eq. (40) by replacing $\Psi_0, \Psi_1, \Psi_2, \Psi_3$ by $\Psi'_0, \Psi'_1, \Psi'_2, \Psi'_3$ and X_0, X_1, X_2, X_3 by X'_0, X'_1, X'_2, X'_3, respectively.

For n = 0, we obtain directly from Eq. (36), the quantity

$$d(0,1,\pm\frac{d_1}{2};0,1,\pm\frac{d_1}{2}) - g(0,1,\pm\frac{d_1}{2};0,1,\pm\frac{d_1}{2}) =$$
$$-g(0,1,\pm\frac{d_1}{2};0,1,\pm\frac{d_1}{2})\Delta^{-1}(0,1,\frac{d_1}{2};0,1,\frac{d_1}{2})A(0,1,\frac{d_1}{2};0,1,\pm\frac{d_1}{2}), \quad (41)$$

From the previous results, we can easily calculate the scattering matrix

$$T(M_m,M_m) = G_1^{-1}(M_m,M_m)\sum_{n=-\infty}^{0}[d(M_m,M_m) - g(M_m,M_m)]G_1^{-1}(M_m,M_m), \quad (42)$$

where $G_1^{-1}(M_m,M_m)$ is given by Eq. (14). $T(M_m,M_m)$ is a 4x4 matrix which can be written as follows

$$T(M_m, M_m) = \begin{pmatrix} T(-\frac{d_1}{2}; -\frac{d_1}{2}) & T(-\frac{d_1}{2}; \frac{d_1}{2}) \\ T(\frac{d_1}{2}; -\frac{d_1}{2}) & T(\frac{d_1}{2}; \frac{d_1}{2}) \end{pmatrix}, \tag{43}$$

Each term $T(\pm\frac{d_1}{2}; \pm\frac{d_1}{2})$ is a 2x2 matrix.

Replacing $G_1(x_3, M_m), G_1(M_m, x_3)$ and $T(M_m, M_m)$ by their respective expressions (Eqs. (17a), (17b), 39 and (43)) and after integration over x_3, we get finally

$$\begin{aligned}
\Delta n_1(\omega^2) = -\frac{\rho_1}{\pi} Im\Big(tr\{ \\
& Q_1^- T(-\frac{d_1}{2}; -\frac{d_1}{2}) Q_1^+ S_1 e^{(-\alpha_1 d_1)} + Q_3^- T(-\frac{d_1}{2}; -\frac{d_1}{2}) Q_3^+ S_3 e^{(-\alpha_3 d_1)} + \\
& [Q_1^- T(-\frac{d_1}{2}; -\frac{d_1}{2}) Q_3^+ + Q_3^- T(-\frac{d_1}{2}; -\frac{d_1}{2}) Q_1^+] S_+ e^{[-(\alpha_1+\alpha_3) d_1/2]} + \\
& d_1 Q_1^- T(-\frac{d_1}{2}; -\frac{d_1}{2}) Q_1^- e^{(-\alpha_1 d_1)} + d_1 Q_3^- T(-\frac{d_1}{2}; \frac{d_1}{2}) Q_3^- e^{(-\alpha_3 d_1)} + \\
& [Q_1^- T(-\frac{d_1}{2}; \frac{d_1}{2}) Q_3^- + Q_3^- T(-\frac{d_1}{2}; \frac{d_1}{2}) Q_1^-] S_- e^{[-(\alpha_1+\alpha_3) d_1/2]} + \\
& d_1 Q_1^+ T(\frac{d_1}{2}; -\frac{d_1}{2}) Q_1^+ e^{-(\alpha_1 d_1)} + d_1 Q_3^- T(\frac{d_1}{2}; -\frac{d_1}{2}) Q_3^+ e^{(-\alpha_3 d_1)} + \\
& [Q_1^+ T(\frac{d_1}{2}; -\frac{d_1}{2}) Q_3^+ + Q_3^+ T(\frac{d_1}{2}; -\frac{d_1}{2}) Q_1^+] S_- e^{[-(\alpha_1+\alpha_3) d_1/2]} + \\
& Q_1^+ T(\frac{d_1}{2}; \frac{d_1}{2}) Q_1^- S_1 e^{-(\alpha_1 d_1)} + Q_3^+ T(\frac{d_1}{2}; \frac{d_1}{2}) Q_3^- S_3 e^{(-\alpha_3 d_1)} + \\
& [Q_1^+ T(\frac{d_1}{2}; \frac{d_1}{2}) Q_1^- + Q_3^+ T(\frac{d_1}{2}; \frac{d_1}{2}) Q_1^-] S_+ e^{[-(\alpha_1+\alpha_3) d_1/2]} \}\Big),
\end{aligned} \tag{44}$$

where

$$S_1 = \frac{sh(\alpha_1 d_1)}{\alpha_1}, \; S_3 = \frac{sh(\alpha_3 d_1)}{\alpha_3}, \tag{45}$$

$$S_- = 2\frac{sh[(\alpha_1 - \alpha_3) d_1/2]}{\alpha_1 - \alpha_3}, \; S_+ = 2\frac{sh[(\alpha_1 + \alpha_3) d_1/2]}{\alpha_1 + \alpha_3}, \tag{46}$$

Following the same procedure as the one presented above for the media 1 of the superlattice, we obtain the variation of the density of states $\Delta_2 n(\omega^2)$ for the media 2 of the semi-infinite superlattice

© 2020 by the authors. Submitted to *Nanomaterials* for possible open access publication under the terms and conditions of the Creative Commons Attribution (CC BY) license (http://creativecommons.org/licenses/by/4.0/).
